# Use of ChAd3-EBO-Z Ebola virus vaccine in Malian and US adults, and boosting of Malian adults with MVA-BN-Filo: a phase 1, single-blind, randomised trial, a phase 1b, open-label and double-blind, dose-escalation trial, and a nested, randomised, double-blind, placebo-controlled trial

**DOI:** 10.1016/S1473-3099(15)00362-X

**Published:** 2016-01

**Authors:** Milagritos D Tapia, Samba O Sow, Kirsten E Lyke, Fadima Cheick Haidara, Fatoumata Diallo, Moussa Doumbia, Awa Traore, Flanon Coulibaly, Mamoudou Kodio, Uma Onwuchekwa, Marcelo B Sztein, Rezwanul Wahid, James D Campbell, Marie-Paule Kieny, Vasee Moorthy, Egeruan B Imoukhuede, Tommy Rampling, Francois Roman, Iris De Ryck, Abbie R Bellamy, Len Dally, Olivier Tshiani Mbaya, Aurélie Ploquin, Yan Zhou, Daphne A Stanley, Robert Bailer, Richard A Koup, Mario Roederer, Julie Ledgerwood, Adrian V S Hill, W Ripley Ballou, Nancy Sullivan, Barney Graham, Myron M Levine

**Affiliations:** a Center for Vaccine Development, University of Maryland School of Medicine, Baltimore, MD, USA; b Centre pour le Développement des Vaccins du Mali, Bamako, Mali, West Africa; c World Health Organization, Geneva, Switzerland; d Jenner Institute and Centre for Clinical Vaccinology and Tropical Medicine, University of Oxford, Oxford, UK; e National Institute for Health Research Oxford Biomedical Research Centre, Oxford, UK; f GlaxoSmithKline Vaccines, Rixensart, Belgium; g The EMMES Corporation, Rockville, MD, USA; h Vaccine Research Center, National Institute of Allergy and Infectious Diseases, Bethesda, MD, USA

## Abstract

**Background:**

The 2014 west African Zaire Ebola virus epidemic prompted worldwide partners to accelerate clinical development of replication-defective chimpanzee adenovirus 3 vector vaccine expressing Zaire Ebola virus glycoprotein (ChAd3-EBO-Z). We aimed to investigate the safety, tolerability, and immunogenicity of ChAd3-EBO-Z in Malian and US adults, and assess the effect of boosting of Malians with modified vaccinia Ankara expressing Zaire Ebola virus glycoprotein and other filovirus antigens (MVA-BN-Filo).

**Methods:**

In the phase 1, single-blind, randomised trial of ChAd3-EBO-Z in the USA, we recruited adults aged 18–65 years from the University of Maryland medical community and the Baltimore community. In the phase 1b, open-label and double-blind, dose-escalation trial of ChAd3-EBO-Z in Mali, we recruited adults 18–50 years of age from six hospitals and health centres in Bamako (Mali), some of whom were also eligible for a nested, randomised, double-blind, placebo-controlled trial of MVA-BN-Filo. For randomised segments of the Malian trial and for the US trial, we randomly allocated participants (1:1; block size of six [Malian] or four [US]; ARB produced computer-generated randomisation lists; clinical staff did randomisation) to different single doses of intramuscular immunisation with ChAd3-EBO-Z: Malians received 1 × 10^10^ viral particle units (pu), 2·5 × 10^10^ pu, 5 × 10^10^ pu, or 1 × 10^11^ pu; US participants received 1 × 10^10^ pu or 1 × 10^11^ pu. We randomly allocated Malians in the nested trial (1:1) to receive a single dose of 2 × 10^8^ plaque-forming units of MVA-BN-Filo or saline placebo. In the double-blind segments of the Malian trial, investigators, clinical staff, participants, and immunology laboratory staff were masked, but the study pharmacist (MK), vaccine administrator, and study statistician (ARB) were unmasked. In the US trial, investigators were not masked, but participants were. Analyses were per protocol. The primary outcome was safety, measured with occurrence of adverse events for 7 days after vaccination. Both trials are registered with ClinicalTrials.gov, numbers NCT02231866 (US) and NCT02267109 (Malian).

**Findings:**

Between Oct 8, 2014, and Feb 16, 2015, we randomly allocated 91 participants in Mali (ten [11%] to 1 × 10^10^ pu, 35 [38%] to 2·5 × 10^10^ pu, 35 [38%] to 5 × 10^10^ pu, and 11 [12%] to 1 × 10^11^ pu) and 20 in the USA (ten [50%] to 1 × 10^10^ pu and ten [50%] to 1 × 10^11^ pu), and boosted 52 Malians with MVA-BN-Filo (27 [52%]) or saline (25 [48%]). We identified no safety concerns with either vaccine: seven (8%) of 91 participants in Mali (five [5%] received 5 × 10^10^ and two [2%] received 1 × 10^11^ pu) and four (20%) of 20 in the USA (all received 1 × 10^11^ pu) given ChAd3-EBO-Z had fever lasting for less than 24 h, and 15 (56%) of 27 Malians boosted with MVA-BN-Filo had injection-site pain or tenderness.

**Interpretation:**

1 × 10^11^ pu single-dose ChAd3-EBO-Z could suffice for phase 3 efficacy trials of ring-vaccination containment needing short-term, high-level protection to interrupt transmission. MVA-BN-Filo boosting, although a complex regimen, could confer long-lived protection if needed (eg, for health-care workers).

**Funding:**

Wellcome Trust, Medical Research Council UK, Department for International Development UK, National Cancer Institute, Frederick National Laboratory for Cancer Research, Federal Funds from National Institute of Allergy and Infectious Diseases.

**Research in context**

**Evidence before this study**

The outbreak of Zaire Ebola virus disease in west Africa in 2014–15 was unparalleled in the morbidity and mortality burden that it evoked, its involvement of health-care and front-line workers, and the intensive transmission documented within urban slums and rural populations. Stakeholder meetings at WHO in August and September, 2014, concluded that clinical development of candidate vaccines to prevent Ebola virus disease should be accelerated. As of mid-August, 2014, one promising candidate, replication-defective chimpanzee adenovirus 3 vector expressing Zaire Ebola virus glycoprotein (ChAd3-EBO-Z), which highly protected non-human primates (NHPs) against lethal challenge with virulent Ebola virus, had not yet been given to human beings. On Aug 30, 2014, and again on July 31, 2015, we searched PubMed for publications (no language or date restrictions) using various combinations of the terms “Ebolavirus”, “vaccine”, “Ebola”, “clinical trials”, “phase 1 clinical trials”, “non-human-primates”, “non-clinical trials”, “chimpanzee adenovirus”, “adenovirus vector vaccines”, and “Modified Vaccinia Ankara vector”. We focused on studies published since 1995, but included older reports where relevant. As of July 31, 2015, only two clinical trials had been published of ChAd3-EBO-Z, one as a bivalent vaccine (1:1 mix with replication-defective chimpanzee adenovirus 3 vector expressing Sudan Ebola virus glycoprotein; US participants) and the other as a monovalent vaccine (UK participants). Both publications represent groups working within the WHO consortium. One publication described preclinical challenge trials in NHPs assessing the short-term (5 weeks after vaccination) and extended (10 months after vaccination) efficacy of the vaccine.

**Added value of this study**

This study constitutes the first phase 1 report of monovalent ChAd3-EBO-Z vaccine in west Africans and compares four doses, consisting of 1 × 10^10^ particle units (pu), 2·5 × 10^10^ pu, 5 × 10^10^ pu, and 1 × 10^11^ pu. The results in both Malian and US participants document that the 1 × 10^11^ pu dose is well tolerated and significantly more immunogenic than are low doses in elicitation of antiglycoprotein antibodies measured with ELISA. 91% of Malian and 60% of US participants given a single dose of ChAd3-EBO-Z attained titres that are associated with protection of NHPs against lethal challenge with wild-type Zaire Ebola virus, suggesting, by extrapolation, that this dose could protect humans against natural infection. A single booster dose of modified vaccinia Ankara expressing Zaire Ebola virus glycoprotein stimulated anamnestic antiglycoprotein antibody and CD4 and CD8 T-cell responses to glycoprotein peptides, suggesting, by extrapolation from results in NHPs, that this booster might extend the duration of high-level protection.

**Implications of all the available evidence**

With optimistic extrapolation of these results, a single 1 × 10^11^ pu dose of ChAd3-EBO-Z might be sufficiently well tolerated and immunogenic to be effective in interruption of transmission of Ebola virus to family members and other close contacts of index patients if used in a ring vaccination tactic after rapid identification of cases. A heterologous prime and boost regimen consisting of a ChAd3-EBO-Z prime followed 2–3 months afterwards by a boost with modified vaccinia Ankara expressing Zaire Ebola virus glycoprotein could confer long-term protection to subgroups (eg, health-care and front-line workers) that need extended protection.

## Introduction

By August 2014, the burgeoning west African Zaire Ebola virus epidemic, which began in rural Guinea and spread to adjacent Liberia and Sierra Leone,^1^ was accelerated by transmission in crowded urban slums. Health-care and other front-line workers accounted for about 5–10% of deaths, and ensuing absenteeism weakened curative and preventive services.^2^, ^3^ Absence of licensed anti-Ebola virus treatments or vaccines to combat the epidemic contributed to a public health calamity in the world's least developed region.^4^ A glimmer of hope came from two Ebola vaccines in development that protected non-human primates (NHPs) against lethal challenge with Ebola virus.^5^, ^6^, ^7^, ^8^ One vaccine, based on a replication-defective chimpanzee adenovirus 3 vector expressing Zaire Ebola virus glycoprotein (ChAd3-EBO-Z),^9^ had never been given to human beings.

In this report, we describe the accelerated clinical development programme that investigated the safety, tolerability, and immunogenicity of ChAd3-EBO-Z at four different doses in Malian adults and two different doses in US adults. Because findings from studies^9^ in NHPs have shown that both immunogenicity and duration of high-level protection against challenge can be extended by administration of a dose of modified vaccinia Ankara (MVA)-encoding Zaire Ebola virus glycoprotein, we sought to assess a heterologous booster in humans primed with ChAd3-EBO-Z. We therefore also describe the effect of boosting of Malians with a heterologous vector, MVA expressing Zaire Ebola virus glycoprotein and other filovirus antigens (MVA-BN-Filo).

## Methods

### Study design and participants

A phase 1b, open-label and double-blind, dose-escalation trial of ChAd3-EBO-Z was initiated at the Center for Vaccine Development (CVD)–Mali, Bamako, Mali, in adults 18–50 years of age, recruited from six hospitals and health centres. A phase 1, single-blind, randomised trial of ChAd3-EBO-Z was also initiated at the CVD in Baltimore, Maryland, USA, in participants aged 18–65 years, recruited from the University of Maryland medical community and the Baltimore community. Full inclusion and exclusion criteria are listed in the appendix. Recognising that administration to NHPs of a booster vaccination with a heterologous viral vector, MVA-expressing Zaire Ebola virus glycoprotein, enhances both humoral and cell-mediated immune responses and extends protection against challenge, we sought to obtain doses of MVA-expressing Zaire Ebola virus glycoprotein to do a nested booster study of the Malian volunteers primed with ChAd3-EBO-Z. Bavarian Nordic (Martinsried, Germany) provided 30 scarce doses of MVA-BN-Filo (which expresses Zaire Ebola virus and Sudan Ebola virus glycoproteins and other filovirus proteins). To distribute this small number of doses and maximise the information obtained, we designed a nested, randomised, double-blind, placebo-controlled booster trial to assess the effect of MVA-BN-Filo versus saline placebo on the immune response.

Participants provided written informed consent. The Malian study was approved by the University of Bamako Faculty of Medicine, Pharmacy and Odontostomatology Ethics Committee, Malian National Ethics Committee, and WHO Ethics Review Committee. The University of Maryland, Baltimore Institutional Review Board approved the Malian and US trials.

### Randomisation and masking

In the US trial, we randomly allocated (1:1) participants to receive 1 × 10^10^ viral particle units (pu) or 1 × 10^11^ pu. The Malian trial was initially designed to test only two doses, 2·5 × 10^10^ pu (group 1) and 5 × 10^10^ pu (group 2). Because this trial was, to our knowledge, the first time that ChAd3-EBO-Z was given to Africans, and only a few people had received the monovalent vaccine weeks earlier (during the week of Sept 17, 2014) at a trial done at the Centre for Clinical Vaccinology and Tropical Medicine at the University of Oxford^10^ (Oxford, UK), and 20 had received bivalent chimpanzee adenovirus 3-vectored Ebola vaccine at the National Institutes of Health (Bethesda, MD, USA; during Sept 2–23, 2014),^11^ we vaccinated the first five Malians in staggered progression after the first five Oxford vaccinees.^10^ Dose escalation to 5 × 10^10^ pu occurred after the data safety monitoring board (DSMB) reviewed 7 day safety data from the recipients of 2·5 × 10^10^ pu. When extra doses became available, after protocol approval, we randomly allocated additional Malians (1:1) to receive 2·5 × 10^10^ pu (group 3B) or 5 × 10^10^ pu (group 3C) in double-blind fashion; open-label groups received 1 × 10^10^ pu (group 3A) or 1 × 10^11^ pu (group 4). In the nested Malian study, we randomly allocated (1:1) Malians primed with ChAd3-EBO-Z to receive MVA-BN-Filo or placebo.

ARB generated randomisation sequences for randomised segments of the Malian trial and for the US trial. In the US trial, randomisation was accomplished through an online database and randomisation software (AdvantageEDC) managed by the EMMES Corporation (Rockville, MD, USA). We used blocked randomisation for both the Malian (block size of six) and US (block size of four) trials, and simple randomisation for the nested Malian study. Clinical staff assigned each enrolled participant a randomisation number from the electronic data entry system that corresponded to a treatment on a computer-generated randomisation list available only to the unmasked study pharmacist (MK) and vaccine administrator.

In the double-blind segments of the Malian trial, study investigators, clinical staff, participants, and immunology laboratory staff were masked. The study pharmacist (MK) and vaccine administrator, who were not involved in other trial assessments, and the study statistician (ARB) were unmasked. In the open-label segments, investigators were aware of dose assignment. In the US trial, study investigators were not masked to study allocation, but participants were masked to the dose of vaccine that they received.

### Procedures

ChAd3-EBO-Z drug substance was manufactured at Advent (Pomezia, Italy), an Okairos (now GlaxoSmithKline) subsidiary, and drug product was vialled at the Vaccine Research Center Vaccine Pilot Plant (Frederick, MD, USA) under contract with the Vaccine Clinical Materials Program, Leidos Biomedical Research (Frederick, MD, USA). The vaccine is a sterile, aqueous, buffered solution that contains ChAd3-EBO-Z in single-dose vials. We stored vaccine at below −60°C. We derived the doses for Malian participants by adjustment of the volume of vaccine injected, as was done in the Oxford trial,^10^ to 110 μL (1 × 10^10^ pu), 275 μL (2·5 × 10^10^ pu), 550 μL (5 × 10^10^ pu), or 1100 μL (1 × 10^11^ pu). For US participants, we delivered the two doses (1 × 10^10^ pu or 1 × 10^11^ pu) in 1·0 mL volume. We gave single doses of vaccine intramuscularly. We injected vaccines into the non-dominant arm triceps.

MVA-BN-Filo vaccine, which encodes Zaire Ebola virus and Sudan Ebola virus glycoproteins, Marburg virus glycoprotein, and Tai-Forest Ebola virus nucleoprotein, was manufactured by Impfstoffwerk Dessau-Torman (Dessau-Roßlau, Germany), and supplied as a liquid formulation in tris and NaCl in 2 mL vials; each 0·5 mL dose contained 3 × 10^8^ plaque-forming units. MVA-BN-Filo was the only MVA-expressing Zaire Ebola virus glycoprotein that was available to boost the Malian participants in a timely manner and, to our knowledge, represents the first use of MVA-BN-Filo in an African population.

We watched participants for immediate-onset adverse (eg, anaphylactic) reactions for 60 min after vaccination. Follow-up visits were on days 7, 14, 28, 90, and 180 after primary or booster vaccination. We also saw Malians on day 1; we contacted US participants by telephone on day 1. We recorded local reactions daily for 7 days after the vaccination and reported unsolicited symptoms for 28 days after vaccination. We reviewed symptoms at each follow-up visit and collected blood for tests to monitor participants' health status, including a full blood count, urea and electrolyte measurements, and liver profile. We did severity grading of adverse events (AEs) and assignment of causal relation of unsolicited AEs according to predefined criteria in the study protocols.

We measured plasma IgG responses to glycoproteins of Zaire Ebola virus (all participants) and Sudan Ebola virus (Malian 1 × 10^11^ pu recipients only) with ELISA.^12^ We expressed chimpanzee adenovirus 3-neutralising and adenovirus 5-neutralising antibodies as inhibitory concentration 90 reciprocal titres.^13^ We quantified Zaire Ebola virus glycoprotein-specific T-cell responses with intracellular cytokine staining.^11^, ^14^ We stimulated cryopreserved peripheral blood mononuclear cells obtained at 0 weeks, 2 weeks, and 4 weeks after ChAd3-EBO-Z vaccination and 0 weeks, 1 week, 2 weeks, and 4 weeks after MVA-BN-Filo boost with overlapping peptide pools for Zaire Ebola virus glycoprotein (Z1 and Z2). We then quantified memory CD4 and CD8 T cells (defined by CD45RA and CCR7 expression patterns) producing interleukin 2, interferon γ, or tumour necrosis factor α (TNFα).^11^, ^14^ We deemed responders participants with CD4 or CD8 responses to any peptide pool (measured by interleukin 2, interferon γ, or TNFα) after vaccination, as described elsewhere.^14^

### Outcomes

The primary outcome was safety, measured with actively (solicited—ie, sought by investigators) collected data for AEs for 7 days after vaccination. The secondary outcome was immunogenicity, assessed with ELISA for antibody responses and an intracellular cytokine staining assay for T-cell responses. We measured baseline antivector antibodies using neutralisation assays. A full list of primary and secondary outcomes is given in the appendix.

### Statistical analysis

The sample size of 91 for the Malian trial balances the need to avoid exposure of a large group to an unknown risk with the need for data from an adequate sample. We based group sample sizes on availability of study product. This sample size should allow establishment of the magnitude of AEs, rather than obtaining of significance for differences between groups. A scarcity of vaccine doses and the desire to study the safety and immunogenicity of a range of dose levels drove sample sizes in the nested trial. In the US trial, the study design was phase 1 dose escalation based on a target accrual of 20 adult participants divided equally between two dose groups.

We reported ELISA antibody responses to Zaire Ebola virus glycoprotein as geometric mean titre (GMT), with 95% CIs. We defined positive responses as significant (α=0·05) increases in log-ELISA from baseline, with use of paired *t* tests.^11^ We used Fisher's exact tests for between-group comparisons of proportions of subjects with positive responses or for responses of more than reference values, Student's *t* tests for the magnitude of the antibody response after log transformation, and Wilcoxon tests to compare the magnitude of T-cell responses or non-normally distributed antibody titres. Tests were two-sided, without multiple comparisons adjustment. We used McNemar's tests to assess whether CD4-positive and CD8-positive T-cell responses occurred in the same or different participants. We analysed associations between antibody and T-cell responses with Spearman's correlation. We fitted a linear regression model to assess associations of prime-boost interval and priming ChAd3-EBO-Z dose with postboost ELISA response. We analysed and displayed T-cell intracellular cytokine staining data with SPICE 5.3.5.^15^ We used SAS 9.3 for other analyses. Analyses were per protocol, but no participants were randomly allocated but not vaccinated and no participants received a product different from their randomisation assignment.

Clinical trial monitoring was provided by CVD–Mali and CVD monitors. A DSMB furnished independent oversight for the Malian trial, whereas a protocol review safety team oversaw the US trial. Both trials are registered with ClinicalTrials.gov, numbers NCT02231866 (US) and NCT02267109 (Malian).

### Role of the funding source

The funders of the study had no role in study design, data collection, data analysis, data interpretation, or writing of the report. The corresponding author had full access to all the data in the study and had final responsibility for the decision to submit for publication.

## Results

**Figure 1:**
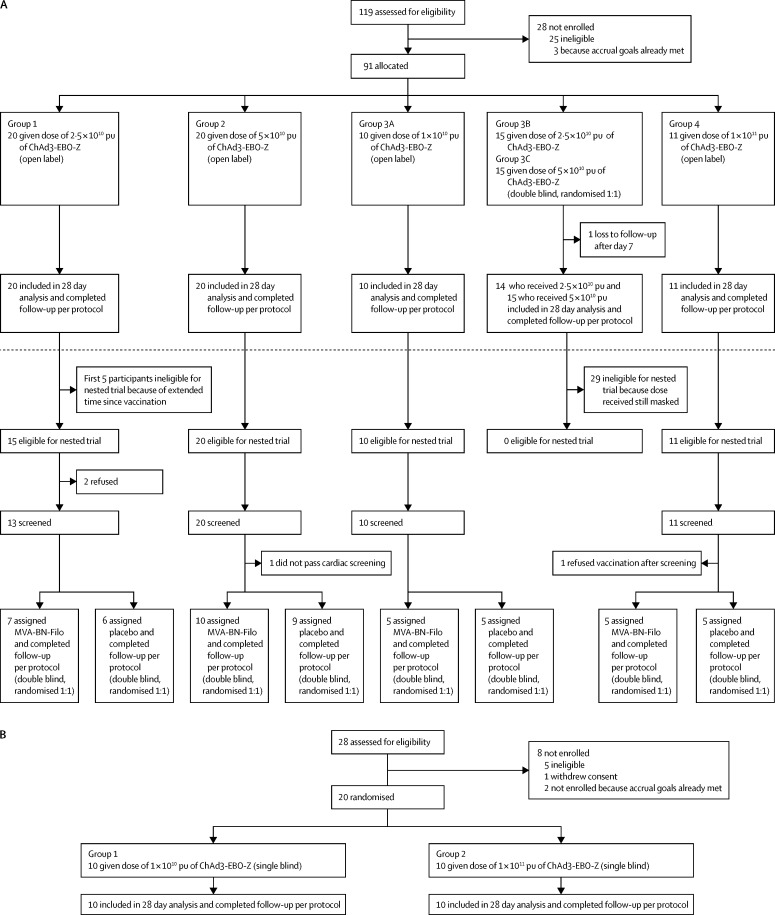
Trial profile (A) Malian trial. (B) US trial. ChAd3-EBO-Z=replication-defective chimpanzee adenovirus 3 vector vaccine expressing Zaire Ebola virus glycoprotein. MVA-BN-Filo=modified vaccinia Ankara expressing Zaire Ebola virus glycoprotein and other filovirus antigens. pu=particle units.

Between Oct 8, 2014, and Oct 23, 2014, 20 (22%) participants (group 1) in Mali received 2·5 × 10^10^ pu of ChAd3-EBO-Z, and between Nov 4, 2014, and Nov 6, 2014, we vaccinated a further 20 (22%) participants (group 2) with 5 × 10^10^ pu (figure 1). Shortly after enrolment of groups 1 and 2 began, 40 additional doses of ChAd3-EBO-Z vaccine became available for use in Mali. Accordingly, after ethical committee approvals and DSMB concurrence, on Nov 10, 2014, we vaccinated ten (11%) additional participants (group 3A) with a dose of 1 × 10^10^ pu, thereby providing a low-dose group for comparison. Between Nov 13, 2014, and Nov 15, 2014, we randomly allocated 30 (33%) additional participants (15 [16%] to each group) to receive a dose of either 2·5 × 10^10^ pu (group 3B) or 5 × 10^10^ pu (group 3C), with double-blind clinical follow-up. Finally, 11 additional doses became available for the Mali trial; with ethical approval of the amended protocol and DSMB concurrence, between Nov 25, 2014, and Nov 26, 2014, 11 (12%) participants received (open label) a 1 × 10^11^ pu dose. Between Nov 10, 2014, and Nov 18, 2014, we vaccinated 20 US participants with ChAd3-EBO-Z (ten [50%] with 1 × 10^10^ pu and ten [50%] with 1 × 10^11^ pu).

Because of the small number of doses of MVA-BN-Filo available, 56 (62%) of the 91 Malians primed with ChAd3-EBO-Z were deemed eligible for the nested trial, in which we randomly allocated them to receive MVA-BN-Filo or placebo. Eligible participants came from groups 1, 2, 3A, and 4: 15 (27%) of 20 participants from group 1 (we excluded the first five vaccinees because they had received their vaccine several weeks earlier than the remaining 15 had because the safety data from these first five participants were reviewed before further vaccinations took place); the 20 (36%) participants from group 2; the ten (18%) participants from group 3A; and the 11 (20%) participants from group 4. The 29 (one was lost to follow-up after day 7) additional vaccinees who had been randomly allocated to receive 2·5 × 10^10^ pu (group 3B) or 5 × 10^10^ pu (group 3C) were ineligible because the dose that they had received was still masked. We had insufficient time to reprogram electronic data capture to accommodate random allocation of a secondary subgroup while preserving masking of the priming dose. Between Feb 9, 2015, and Feb 16, 2015, we enrolled 52 (93%) of these 56 eligible participants (three declined to participate and one was medically ineligible based on cardiac screening criteria), representing all four ChAd3-EBO-Z doses and a fairly tight range of intervals (79–111 days) since priming immunisation with ChAd3-EBO-Z, who we randomly allocated to receive MVA-BN-Filo (27 [52%] participants) or saline placebo (25 [48%] participants), followed up clinically in double-blind fashion.

Analysis of viral titre in vials from the vaccine lot used in the clinical trials showed 1·0 × 10^11^ pu/mL when tested at the National Institutes of Health (Bethesda, MD, USA) and 9·1 × 10^10^ pu/mL when tested at the Jenner Institute, University of Oxford (Oxford, UK). Table 1 summarises participants' demographic and other characteristics; all Malians were health-care and front-line workers.

Table 2 summarises the frequency and severity of solicited AEs (unsolicited—ie, offered voluntarily by participants—AEs described in appendix), by dose, for 7 days after ChAd3-EBO-Z vaccination or MVA-BN-Filo booster (primary outcome). Most AEs were mild, with no unexpected serious adverse reactions suspected. One (1%) serious AE in Mali (tuberculous peritonitis; received 2·5 × 10^10^ pu) was unrelated to vaccine. The predominant solicited AE was fever, occurring in seven (8%) of 91 Malian (five [5%] received 5 × 10^10^ pu and two [2%] received 1 × 10^11^ pu) and four [20%] of 20 US (all received 1 × 10^11^ pu) participants; ten of 11 fevers resolved by 24 h after vaccination and none persisted for longer than 24 h. One (1%) fever of 37·6°C occurred in a Malian volunteer on day 2 after vaccination (5 × 10^10^ pu), but still resolved within 24 h. One (1%) Malian (5 × 10^10^ pu) and two (10%) US (1 × 10^11^ pu) vaccinees had fevers with temperatures higher than 38·5°C, accompanied by systemic symptoms (eg, fatigue, myalgia, arthralgia, headache, chills, or nausea). The appendix summarises laboratory abnormalities noted up to day 28. Most episodes (ten of 11) of the most frequent abnormality, lymphopenia, occurred on day 1 after vaccination and self-resolved. In the Malian participants, we noted single cases of moderate (109 × 10^9^/L; 5 × 10^10^ pu) and severe (63 × 10^9^/L; 2·5 × 10^10^) asymptomatic thrombocytopenia on day 1 after vaccination that resolved by day 7.

AEs were uncommon in the 27 participants boosted with MVA-BN-Filo. By day 7, the most common local reactions were injection-site pain or tenderness (table 2). Two (7%) participants boosted with MVA-BN-Filo developed mild fever and associated injection-site pain, mild myalgia, headache, and fatigue. Thick smears for malaria parasites were negative, and both were well by day 3. Other AEs noted up to day 28 are summarised in the appendix. Of 25 participants allocated to placebo booster, one (4%) had an isolated mild fever of 37·9°C.

**Figure 2:**
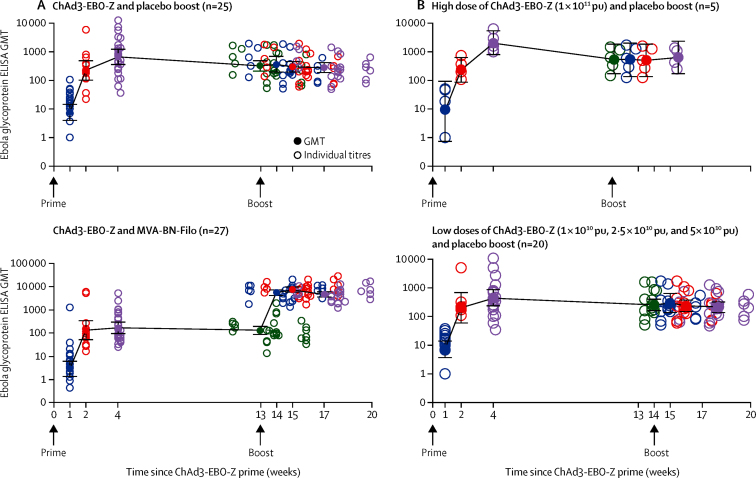
Anti-Zaire Ebola virus glycoprotein ELISA titres (background subtracted) for the Malian participants in the nested MVA-BN-Filo booster trial Titres of individual participants are plotted as open circles at the actual week after priming immunisation with ChAd3-EBO-Z vaccine. GMTs are plotted as filled circles with associated 95% CIs. For participants in the booster study, their boost visits ranged from 11·2 weeks to 15·8 weeks after priming; the GMT for the boost visit is plotted at the median of 13 weeks, and the GMTs for postboost visits are plotted at the subsequent target intervals (ie, 1 week, 2 weeks, and 4 weeks after boosting). (A) Participants primed with ChAd3-EBO-Z and then boosted with saline placebo are compared with those primed with ChAd3-EBO-Z and then boosted with MVA-BN-Filo. (B) The longevity of the antiglycoprotein response in participants vaccinated with the 1 × 10^11^ pu high dose of ChAd3-EBO-Z and boosted with saline placebo is shown and compared with the magnitude of response of the participants who received low doses (1 × 10^10^ pu, 2·5 × 10^10^ pu, or 5 × 10^10^ pu) of ChAd3-EBO-Z, followed by a saline booster. ChAd3-EBO-Z=replication-defective chimpanzee adenovirus 3 vector vaccine expressing Zaire Ebola virus glycoprotein. GMT=geometric mean titre. MVA-BN-Filo=modified vaccinia Ankara expressing Zaire Ebola virus glycoprotein and other filovirus antigens. pu=particle units.

We measured plasma IgG responses to Zaire Ebola virus glycoprotein with ELISA and compared them with titres that conferred protection in NHP efficacy trials (secondary outcome).^5^ Table 3 and the appendix display the proportion of ChAd3-EBO-Z vaccinees in each group who showed serological responses by day 28, and the GMT. We noted high serological response rates for all dose groups, but GMT was significantly higher in recipients of 1 × 10^11^ pu than in those given low doses (1 × 10^10^ pu, 2·5 × 10^10^ pu, and 5 × 10^10^ pu). The proportion of Malian vaccinees with day 28 reciprocal titres of 500 or higher, 1000 or higher, or 1500 or higher was significantly higher in recipients of the high dose than in those given low vaccine doses (table 3, figure 2). After vaccination with ChAd3-EBO-Z, the GMT peaked at 28 days and fell only slowly through the next 12 weeks. After boosting with MVA-BN-Filo, the GMT rapidly rose by 36 times and persisted at this high level. The antiglycoprotein antibody persisted at a much higher level in recipients of the high dose of ChAd3-EBO-Z than in those of the low doses (appendix).

The participants in the booster study did not differ from the non-boosted participants in terms of baseline characteristics (table 1), and the MVA-BN-Filo booster recipients were not different from the saline controls with respect to age, ChAd3-EBO-Z priming dose, or prime-boost interval (MVA-BN Filo mean interval 13·8 weeks [SD 0·29]; placebo 13·7 weeks [0·29]). Serological responses of the Malian MVA-BN-Filo booster participants are summarised in table 4, figure 2, and the appendix). The GMT 28 days after boosting (9279·6 [95% CI 7193·2–11 971·2]) was significantly higher than that 28 days after priming (356·4 [207·3–612·6]) for the 27 participants boosted with MVA-BN-Filo (geometric mean-fold increase of 26·0 [14·6–46·3]; p<0·0001); the rise to 28 days after boosting compared with preboost GMT (276·0 [183·0–416·4]) was similarly significant (geometric mean-fold increase of 33·6 [22·8–49·6]; p<0·0001). The five Malian participants who received 1 × 10^11^ pu of ChAd3-EBO-Z and were subsequently boosted with MVA-BN-Filo offered an opportunity to assess whether ChAd3-EBO-Z had primed these individuals to mount accelerated serological responses to heterologous Sudan Ebola virus glycoprotein following the boost. 1 week after the boost, four (80%) of these five participants exhibited serological responses to the Sudan Ebola virus glycoprotein (appendix). By contrast, only one (20%) of these five showed a minimum serological response to the distantly related (different genus) Marburg virus glycoprotein after the boost (data not shown).

The durability of the antibody response to Zaire Ebola virus glycoprotein after administration of high-dose (1 × 10^11^ pu) ChAd3-EBO-Z alone was assessed in the five recipients of this dose who subsequently received saline booster and provided plasma specimens 180 days after the boost—ie, 259 days after priming (secondary outcome, appendix). Even after 259 days, we noted only a very shallow slope of decay of antibody titres, showing impressive longevity of the antibody response after administration of a single high dose of ChAd3-EBO-Z.

The appendix shows the progressive flow cytometry gating strategy used to enumerate antigen-specific T cells. T-cell responses to primary immunisation with ChAd3-EBO-Z and after MVA-BN-Filo boosting are summarised in the appendix (secondary outcome). T-cell responses after priming were slight and of small magnitude: 15 (31%) of 49 participants showed either positive CD4 or CD8 responses after priming with ChAd3-EBO-Z, including eight (16%) who mounted measurable CD8 T-cell responses and 11 (22%) who showed CD4 T-cell responses. We noted a strong association between CD4 and CD8 responses in individual participants, except at 7 days after the MVA-BN-Filo boost, when a higher proportion of the 25 individuals had a CD8 response than had a CD4 response (two [8%] CD4-negative and CD8-negative, seven [28%] CD4-negative and CD8-positive, and 16 [64%] CD4-positive and CD8-positive; McNemar's test p=0·0082). Cell-mediated immunity responses after priming with ChAd3-EBO-Z were stable and long-lived, as shown over time for the 25 participants primed with ChAd3-EBO-Z who later received a placebo booster. By contrast, of the 27 participants boosted with MVA-BN-Filo, we noted high-magnitude postboost CD4 and CD8 responses in 23 (85%) participants (appendix). Similar to after priming, we noted a high degree of concordance between participants who showed both CD4 and CD8 responses. Most Zaire Ebola virus glycoprotein-specific CD8 memory T cells were multifunctional, producing both interferon γ and TNFα, or all three cytokines (interferon γ, TNFα, and interleukin 2); 25% of CD8 T cells were interferon γ single-positive.

## Discussion

Our results identified the ChAd3-EBO-Z dose for large-scale manufacture of the formulation slated for phase 2 and 3 trials in Africa. A single 1 × 10^11^ pu dose of ChAd3-EBO-Z elicited strong antiglycoprotein antibody responses in all participants. Glycoprotein-specific antibodies elicited by adenovirus 5 and chimpanzee adenovirus 3-vectored vaccines constitute a non-mechanistic correlate of protection against otherwise lethal Ebola virus challenge to NHPs,^5^, ^9^, ^16^ although T cells seem key in mediation of protection conferred by adenovirus-vectored and DNA Ebola vaccines.^5^, ^17^ Of NHPs immunised with adenovirus 5-vectored Ebola vaccine and challenged about 1 month later,^5^, ^9^ animals that attained reciprocal titres of 1000 or higher (with use of ELISA as described by Sullivan and colleagues^5^) showed 77% vaccine efficacy against death. Similarly, NHPs that were vaccinated with single-dose chimpanzee adenovirus 3-vectored Zaire Ebola virus vaccine and attained reciprocal titres of 967 or higher were 100% protected against lethal challenge at 5 weeks.^9^ Thus, the finding that 90·9% of Malian recipients of 1 × 10^11^ pu attained reciprocal titres of 1000 or higher is encouraging, generating optimism that this vaccine dose might confer high-level protection to vaccinated human beings in the field, at least in the short term. Many believe that the level of infective inocula to which humans are naturally exposed is much less than that of parenteral inocula given to NHPs. If so, ChAd3-EBO-Z could prove useful for diminishing of transmission in defined target groups in future Ebola outbreaks. In NHPs, Zaire Ebola virus glycoprotein-specific CD8 memory T cells expressing both interferon γ and TNFα were associated with short-term protection (5 week challenge) after one dose of ChAd3-EBO-Z, whereas CD8 memory T cells positive for all three cytokines after booster with MVA-BN-Filo were associated with extended protection (10 month challenge).^9^

Subpopulations at high risk of transmission of Ebola virus in Africa consist of two distinct categories. The first consist of family members, neighbours, and other close contacts with people with confirmed Ebola cases. The second includes health-care and other front-line workers, and people who perform ritual funeral practices for patients who have died from Ebola. High-coverage, concentric, single-dose containment ring vaccination with immunogenic Ebola vaccines for people in and around the households of people with index Ebola cases could diminish transmission between family members, neighbours, and known contacts, as was achieved with the surveillance and containment strategy that interrupted smallpox transmission in west Africa.^18^, ^19^ For reactive vaccination to successfully contain Ebola virus disease outbreaks, a logistically practical, single-dose regimen that confers high-level efficacy (even if in the short term) and enables high coverage is needed. The precedent for this strategy was established with the replicating recombinant vesicular stomatitis virus vector expressing Zaire Ebola virus glycoprotein (rVSV-ZEBOV) vaccine in a phase 3 field trial of efficacy in Guinea.^20^ Within a few days of laboratory confirmation of an Ebola case, a cluster of all contacts and of their contacts was defined, and the cluster was randomly allocated to either ring vaccination of consenting contacts around the case to begin immediately or only after a 21 day delay.^20^ The primary per-protocol analysis, which compared the incidence of laboratory-confirmed Ebola in eligible vaccinated contacts in immediate ring vaccination clusters with that in those in delayed ring vaccination clusters, was limited to confirmed cases that had an onset of illness 10 days or longer after randomisation. Contacts of confirmed cases were significantly protected if ring vaccination with rVSV-ZEBOV was begun immediately rather than after a 21 day delay.^20^ We expect that if ring vaccination was used with high-dose ChAd3-EBO-Z, the vaccine would be similarly effective.

A comparison would be interesting of the ELISA antibody responses to Zaire Ebola virus glycoprotein shown by the Malians who received 1 × 10^11^ pu of ChAd3-EBO-Z with those of Africans who received 2 × 10^7^ plaque-forming units of rVSV-ZEBOV, as used in the Guinea ring vaccination field trial.^20^ So far, no data have been published for immune responses with this dose in Africans to allow a comparison. Data exist for serological responses of participants in Gabon and Kenya who received a dose of rVSV-ZEBOV one log lower than those in the Guinea field trial; however, the ELISA methods of measurement of antiglycoprotein were different to those used to obtain the data in the Malian trial described in this report.^21^ Publication of results of the phase 2 safety and immunogenicity component of the Partnership for Research on Ebola Vaccines in Liberia trial (NCT02344407) might provide data for this point.

Health-care, front-line, and funeral workers who have repetitive exposures to Ebola for extended periods need a vaccination regimen that confers more durable protection than what is expected to be derived from ChAd3-EBO-Z alone (at least on the basis of NHP studies).^9^ Fortunately, accessibility of these individuals makes delivery of a complex vaccination regimen (two spaced doses of two different vaccines) feasible. Accordingly, boosting of front-line workers with heterologous MVA vector expressing Ebola virus glycoprotein could extend the duration of high-level protection, as was noted in NHPs.^9^ Antibody and T-cell responses of Malians boosted with MVA-BN-Filo show its powerful boosting capacity.

When these trials began, the scarcity of vaccine doses constrained trial design options. Some consortium partners proposed maximising the potential number of doses with testing of low doses containing 1 × 10^10^ pu, 2·5 × 10^10^ pu, and 5 × 10^10^ pu.^10^ Results with a chimpanzee adenovirus 3-vectored hepatitis C vaccine with use of such doses provided a rationale.^22^ Other partners argued for testing of two widely separated doses (1 × 10^10^ and 1 × 10^11^ pu) as a starting point to guide further studies.^11^ In this study, we tested all four doses of ChAd3-EBO-Z, once sufficient vaccine doses became available. This strategy acknowledged the possibility that west Africans, differing in genetic background, nutritional state, socioeconomic level, and past exposure to adenoviruses and other pathogens, might mount immunological responses distinct from those of consortium participants from Europe and North America who were 80–90% white and living in affluence. This strategy proved fortuitous because we documented immunological superiority of 1 × 10^11^ pu to other doses. Importantly, adverse reactions were uncommon at this dose. No Malians and only two of ten US recipients of 1 × 10^11^ pu had fever with a temperature of 38·6°C or higher, and none had one with a temperature of 39·6°C or higher. Short-lived fevers will not interfere with ring vaccination.

The results reported in this study paved the way for phase 2 trials in adults (PACTR201504001092179) and children (EudraCT 2014-004714-28) and for a phase 3 efficacy trial in Guinea with the 1 × 10^11^ pu dose of ChAd3-EBO-Z to follow completion of testing of VSV-ZEBOV in the *Ebola ça Suffit* trial.^23^ The partners that collectively managed the field trial operation in Guinea decided to proceed with a second round of testing, and ChAd3-EBO-Z was the vaccine selected on the basis of different criteria, which were an acceptable safety profile, induction of appropriate immune responses in human beings, protection of NHPs, and the timely availability of sufficient vaccine doses. However, evidence of efficacy noted with VSV-ZEBOV in the field trial in Guinea led the DSMB for that trial to decline a switch to assessment of ChAd3-EBO-Z vaccine in the same study design of immediate versus delayed ring vaccination of contacts surrounding cases and instead to continue ring vaccination with VSV-ZEBOV, but only with use of immediate ring vaccination after confirmation of cases; the delayed ring vaccinations that provided the comparator to estimate vaccine efficacy were discontinued. Thus, the efficacy trial transitioned to an assessment of the practicality and logistics of immediate ring vaccination as a control measure used in a west African setting. Indeed, since that DSMB decision, too few cases of Ebola have been confirmed in Guinea to have an efficacy evaluation and, in mid-September, 2015, Guinea had the first Ebola-free week since the previous 12 months.^24^

The timeframe in which the ChAd3-EBO-Z vaccine progressed from preclinical status to a phase 1b trial in Mali was astoundingly short. Ethics committees and the DSMB did their reviews and regulatory authorities arranged vaccine importations rapidly. The first vaccination occurred on Oct 8, 2014 (less than 2 months after the consortium assembled), the last participant was vaccinated on Nov 26, 2014, and the last day 28 blood was drawn on Dec 24, 2014. This short timeframe included obtaining of approvals for several changes in the size and design of the trial as further doses of vaccine became available.

MVA-BN-Filo booster vaccine given 11–16 weeks after priming with ChAd3-EBO-Z was well tolerated and powerfully immunogenic in elicitation of both anamnestic antibody responses and robust multifunctional CD4 and CD8 memory T-cell responses. On the basis of data from NHP challenges,^9^ ChAd3-EBO-Z prime followed by boost with MVA encoding Zaire Ebola virus glycoprotein would be the preferred regimen for immunisation of front-line workers who need long-lived, high-level protection against repetitive exposures. Nevertheless, complexities exist from the perspective of immunisation programmes in procural and delivery of a two-dose schedule with two different vaccines.

This study has limitations. Like other phase 1 trials of Ebola vaccines in Africa,^21^, ^25^ our study had an under-representation of women. Also, our study would have been improved if more Malian participants could have received the 1 × 10^11^ pu dose of ChAd3-EBO-Z than the number that did receive that dose in this study, had more doses been available. Similarly, the small number of doses of MVA-BN-Filo available limited the size of the nested booster study. Finally, our absence of access to a monovalent MVA expressing only Zaire Ebola virus glycoprotein prevented us from also studying that product in the booster study.

**This online publication has been corrected. The corrected version first appeared at thelancet.com/infection on Dec 14, 2015**

## Figures and Tables

**Table 1:** Baseline characteristics

		**Malian adults**								**US adults**		
		Phase 1b trial					Nested booster study			Phase 1 trial		
		1 × 10^10^ pu of ChAd3-EBO-Z (n=10)	2·5 × 10^10^ pu of ChAd3-EBO-Z (n=35)	5 × 10^10^ pu of ChAd3-EBO-Z (n=35)	1 × 10^11^ pu of ChAd3-EBO-Z (n=11)	Overall (n=91)	Boosted with MVA-BN-Filo (n=27)	Boosted with saline placebo (n=25)	Overall (n=52)	1 × 10^10^ pu of ChAd3-EBO-Z (n=10)	1 × 10^11^ pu (n=10)	Overall (n=20)
Sex												
	Male	10 (100%)	29 (83%)	30 (86%)	4 (36%)	73 (80%)	19 (70%)	20 (80%)	39 (75%)	4 (40%)	5 (50%)	9 (45%)
	Female	0	6 (17%)	5 (14%)	7 (64%)	18 (20%)	8 (30%)	5 (20%)	13 (25%)	6 (60%)	5 (50%)	11 (55%)
Age (years)		33·7 (6·8)	35·9 (6·0)	33·7 (6·9)	31·0 (7·1)	34·2 (6·8)	33·6 (5·4)	34·6 (8·3)	34·1 (6·9)	32·9 (12·2)	37·6 (10·4)	35·3 (11·3)
Race												
	Asian	0	0	0	0	0	0	0		0	2 (20%)	2 (10%)
	Black	10 (100%)	35 (100%)	35 (100%)	11 (100%)	91 (100%)	27 (100%)	25 (100%)	52 (100%)	2 (20%)	2 (20%)	4 (20%)
	White	0	0	0	0	0	0	0	0	8 (80%)	6 (60%)	14 (70%)
	Hispanic or Latino	0	0	0	0	0	0	0	0	1	0	1 (5%)
Body-mass index (kg/m^2^)		23·5 (3·4)	26·4 (4·3)	24·6 (4·2)	29·6 (7·4)	25·6 (4·9)	25·6 (6·0)	25·6 (4·4)	25·6 (5·2)	28·6 (5·0)	24·6 (3·4)	26·6 (4·6)

Data are n (%) or mean (SD). pu=particle units. ChAd3-EBO-Z=replication-defective chimpanzee adenovirus 3 vector vaccine expressing Zaire Ebola virus glycoprotein. MVA-BN-Filo=modified vaccinia Ankara expressing Zaire Ebola virus glycoprotein and other filovirus antigens.

**Table 2:** Adverse events

		**Malian adults**								**US adults**		
		Phase 1b trial					Nested booster study			Phase 1 trial		
		1 × 10^10^ pu of ChAd3-EBO-Z (n=10)	2·5 × 10^10^ pu of ChAd3-EBO-Z (n=35)	5 × 10^10^ pu of ChAd3-EBO-Z (n=35)	1 × 10^11^ pu of ChAd3-EBO-Z (n=11)	Overall (n=91)	Boosted with MVA-BN Filo (n=27)	Boosted with saline placebo (n=25)	Overall (n=52)	1 × 10^10^ pu of ChAd3-EBO-Z (n=10)	1 × 10^11^ pu of ChAd3-EBO-Z (n=10)	Overall (n=20)
Local*												
Pain and tenderness												
	Mild	3 (30%)	13 (37%)	14 (40%)	8 (73%)	38 (42%)	13 (48%)	0	13 (25%)	4 (40%)	4 (40%)	8 (40%)
	Moderate	0	0	0	1 (9%)	1 (1%)	2 (7%)	0	2 (4%)	0	0	0
**Systemic**												
Fever (oral temperature)												
	37·6–38·5°C	0	0	4 (11%)	2 (18%)	6 (7%)	2 (7%)	1 (4%)	3 (6%)	0	2 (20%)	2 (10%)
	38·6–39·5°C	0	0	1 (3%)	0	1 (1%)	0	0	0	0	2 (20%)	2 (10%)
Fatigue												
	Mild	2 (20%)	10 (29%)	10 (29%)	3 (27%)	25 (27%)	12 (44%)	0	12 (23%)	3 (30%)	5 (50%)	8 (40%)
	Moderate	0	1 (3%)	2 (6%)	0	3 (3%)	0	0	0	0	2 (20%)	2 (10%)
Myalgia												
	Mild	0	4 (11%)	3 (9%)	1 (9%)	8 (9%)	7 (26%)	0	7 (13%)	1 (10%)	2 (20%)	3 (15%)
	Moderate	0	0	2 (6%)	0	2 (2%)	0	0	0	0	3 (30%)	3 (15%)
Arthralgia												
	Mild	0	2 (6%)	1 (3%)	2 (18%)	5 (5%)	3 (11%)	0	3 (6%)	†	†	†
	Moderate	0	0	2 (6%)	0	2 (2%)	0	0	0	†	†	†
Headache												
	Mild	5 (50%)	14 (40%)	6 (17%)	4 (36%)	29 (32%)	13 (48%)	3 (12%)	16 (31%)	4 (40%)	5 (50%)	9 (45%)
	Moderate	0	1 (3%)	2 (6%)	0	3 (3%)	1 (4%)	0	1 (2%)	1 (10%)	2 (20%)	3 (15%)
Chills												
	Mild	0	1 (3%)	4 (11%)	1 (9%)	6 (7%)	1 (4%)	0	1 (2%)	0	1 (10%)	1 (5%)
	Moderate	0	0	1 (3%)	0	1 (1%)	1 (4%)	0	1 (2%)	0	2 (20%)	2 (10%)
Nausea												
	Mild	0	1 (3%)	1 (3%)	1 (9%)	3 (3%)	1 (4%)	0	1 (2%)	0	0	0
	Moderate	0	0	0	0	0	0	0	0	0	1 (10%)	1 (5%)
**Haematological**												
Lymphopenia												
	750–1000 cells per μL	0	4 (11%)	3 (9%)	3 (27%)	10 (11%)	2 (7%)	0	2 (4%)	3 (30%)	0	3 (15%)
	500–749 cells per μL	0	0	1 (3%)	1 (9%)	2 (2%)	1 (4%)	0	1 (2%)	0	0	0

Data are n (%). The maximum solicited local and systemic reactogenicity symptoms are depicted through to day 7 after vaccination. pu=particle units. ChAd3-EBO-Z=replication-defective chimpanzee adenovirus 3 vector vaccine expressing Zaire Ebola virus glycoprotein. MVA-BN-Filo=modified vaccinia Ankara expressing Zaire Ebola virus glycoprotein and other filovirus antigens.

* Local adverse events were pain, erythema, induration and swelling, pruritus, and warmth. We noted one episode of mild induration and swelling in a Malian participant (1 × 10^11^ pu). We recorded no erythema events.

† Signs or symptoms that were not collected.

**Table 3:** Serological responses after replication-defective chimpanzee adenovirus 3 vector vaccine expressing Zaire Ebola virus glycoprotein vaccination

		**Malian adults**				**US adults**	
		1 × 10^10^ pu (n=10)	2·5 × 10^10^ pu (n=34)*	5 × 10^10^ pu (n=35)	1 × 10^11^ pu (n=11)	1 × 10^10^ pu (n=10)	1 × 10^11^ pu (n=10)
Positive response by day 28		10 (100·0% [69·2–100·0])	33 (97·1% [84·7–99·9])	29 (82·9% [66·4–93·4])	11 (100% [71·5–100·0])	10 (100% [69·2–100·0])	10 (100% [69·2–100·0])
Day 28 geometric titre		295·0 (114·8–758·2)	220·4 (155·9–311·6)	466·0 (289·1–750·9)	1446·9 (759·4–2756·8)	531·5 (249·4–1132·5)	1255·9 (379·7–4154·2)
Day 28 reciprocal titres							
	≥500	3 (30·0% [6·7–65·2])	5 (14·7% [5·0–31·1])	15 (42·9% [26·3–60·6])	10 (90·9% [58·7–99·8])	5 (50·0% [18·7–81·3])	7 (70·0% [34·8–93·3])
	≥1000	1 (10·0% [0·3–44·5])	3 (8·8% [1·9–23·7])	7 (20·0% [8·4–36·9])	10 (90·9% [58·7–99·8])	3 (30·0% [6·7–65·2])	6 (60·0% [26·2–87·8])
	≥1500	1 (10·0% [0·3–44·5])	2 (5·9% [0·7–19·7])	6 (17·1% [6·6–33·6])	6 (54·5% [23·4–83·3])	3 (30·0% [6·7–65·2])	6 (60·0% [26·2–87·8])
Baseline reciprocal titre ≥200							
	Chimpanzee adenovirus 3-neutralising antibody	2 (20·0% [2·5–55·6])	4 (11·8% [3·3–27·5])	3 (8·6% [1·8–23·1])	0 (0% [0–28·5])	..	..
	Adenovirus 5-neutralising antibody	6 (60·0% [26·2–87·8])	30 (88·2% [72·6–96·7])	30 (85·7% [69·7–95·2])	8 (72·7% [39·0–94·0])	..	..

Data are n (% [95% CI]) or mean (95% CI). This table with comparisons with other studies is provided in the appendix. pu=particle units.

* One loss to follow-up after day 7.

**Table 4:** Serological responses after ChAd3-EBO-Z and MVA-BN-Filo or saline placebo vaccinations

	**Malian adults after ChAd3-EBO-Z**					**Malians adults after MVA-BN-Filo**	
	1 × 10^10^ pu (n=10)	2·5 × 10^10^ pu (n=13)	5 × 10^10^ pu (n=19)	1 × 10^11^ pu (n=10)	Overall (n=52)	1 × 10^8^ PFU (n=27)	Saline placebo (n=25)*
**Positive response by**							
Day 7	2 (20·0% [2·5–55·6])	3 (23·1% [5·0–53·8])	2 (10·5% [1·3–33·1])	0 (0% [0–30·8])	7 (13·5% [5·6–25·8])	27 (100% [87·2–100·0])	21 (87·5% [67·6–97·3])
Day 28	10 (100% [69·2–100·0])	13 (100% [75·3–100·0])	18 (94·7% [74·0–99·9])	10 (100% [69·2–100·0])	51 (98·1% [89·7 to 100·0])	27 (100% [87·2–100·0])	20 (83·3% [62·6–95·3])
**Geometric mean titre**							
Day 7	16·2 (8·2–32·2)	6·4 (3·0–13·6)	4·7 (1·7–13·2)	6·5 (1·9–21·7)	..	11 209·3 (8552·6–14 691·4)	341·3 (182·2–637·1)
Day 28	295·0 (114·8–758·2)	204·6 (99·9–423·5)	555·8 (282·2–1094·6)	1493·6 (727·6–3065·9)	..	9279·6 (7193·2–11 971·2)	261·3 (173·9–392·7)
**Day 7 reciprocal titres**							
≥500	0 (0% [0–30·8])	0 (0% [0–24·7])	1 (5·3% [0·1–26·0])	0 (0% [0–30·8])	1 (1·9% [0·0–10·2])	27 (100% [87·2–100·0])	7 (29·2% [12·6–51·0])
≥1000	0 (0% [0–30·8])	0 (0% [0–24·7])	1 (5·3% [0·1–26·0])	0 (0% [0–30·8])	1 (1·9% [0·0–10·2])	27 (100% [87·2–100·0])	5 (20·1% [7·1–42·2])
**Day 28 reciprocal titres**							
≥500	3 (30·0% [1·6–58·4])	2 (15·4% [1·9–45·4])	8 (42·1% [20·6–66·5])	9 (90·0% [55·5–99·7])	22 (42·3% [28·7–56·8])	27 (100% [87·2–100·0])	6 (25·0% [9·8–46·7])
≥1000	1 (10% [0·2–4·5])	2 (15·4% [1·9–45·4])	3 (15·8% [3·4–39·6])	9 (90·0% [55·5–99·7])	15 (28·8% [17·1–43·1])	27 (100% [87·2–100·0])	3 (12·5% [2·7–32·4])
≥1500	1 (10% [0·2–4·5])	1 (7·7% [0·2–36·0])	3 (15·8% [3·4–39·6])	6 (60·0% [26·2–87·8])	11 (21·2% [11·1–34·7])	27 (100% [87·2–100·0])	0 (0% [0–14·2])

Data are n (% [95% CI]) or mean (95% CI). This table with comparisons with other studies is provided in the appendix. ChAd3-EBO-Z=replication-defective chimpanzee adenovirus 3 vector vaccine expressing Zaire Ebola virus glycoprotein. MVA-BN-Filo=modified vaccinia Ankara expressing Zaire Ebola virus glycoprotein and other filovirus antigens. pu=particle units. PFU=plaque-forming units.

* One participant did not provide a blood specimen on day 7 and one did not provide one on day 28.
