# Supplementary material for: Use of ChAd3-EBO-Z Ebola virus vaccine in Malian and US adults, and boosting of Malian adults with MVA-BN-Filo: a phase 1, single-blind, randomised trial, a phase 1b, open-label and double-blind, dose-escalation trial, and a nested, randomised, double-blind, placebo-controlled trial
Source: Lancet Infect Dis. 2016 Jan;16(1):31–42. doi: 10.1016/S1473-3099(15)00362-X (PMC4700389; doi:10.1016/S1473-3099(15)00362-X)
Supplement: Supplementary appendix [file mmc1.pdf]

THE LANCET Infectious Diseases

Supplementary webappendix

This webappendix formed part of the original submission and has been peer reviewed. We post it as supplied by the authors.

Supplement to: Tapia MD, Sow SO, Lyke KE, et al. Use of ChAd3-EBO-Z Ebola virus vaccine in Malian and US adults, and boosting of Malian adults with MVA-BN-Filo: a phase 1, single-blind, randomised trial, a phase 1b, open-label and double-blind, randomised, dose-escalation trial, and a nested, randomised, double-blind, placebo-controlled trial. *Lancet Infect Dis* 2015; published online Nov 3. http://dx.doi.org/10.1016/S1473-3099(15)00362-X.

Supplementary Webappendix

This webappendix has been provided by authors as part of the original submission and is peer reviewed .We post it as supplied by the authors to give readers additional information about the work.

Supplement to: Tapia MD*, Sow SO*, Lyke KE* *(*co-1st authors)* et al. "Safety and immunogenicity trials in Malian (Phase 1b) and U.S. (Phase 1) adults of non-replicating chimpanzee adenovirus vector expressing *Zaire ebolavirus* glycoprotein and a nested randomized, double-blind, placebo-controlled trial to assess the effect of boosting Malian participants with heterologous live vector MVA-BN®-Filo or saline".

“Safety and immunogenicity trials in Malian (Phase 1b) and U.S. (Phase 1) adults of non-replicating chimpanzee adenovirus vector expressing *Zaire ebolavirus* glycoprotein and a nested randomized, double-blind, placebo-controlled trial to assess the effect of boosting Malian participants with heterologous live vector MVA-BN®-Filo or saline”

Milagritos D. Tapia, M.D., [1,2*] Samba O. Sow, M.D.,[2*] Kirsten E. Lyke, M.D.,[1*] Fadima Cheick Haidara, M.D.,[2] Fatoumata Diallo, M.D.,[2] Moussa Doumbia, M.D.,[2] Awa Traore, Pharm.D.,[2] Flanon Coulibaly, M.D.,[2] Mamoudou Kodio, Pharm.D.,[2] Uma Onwuchekwa, B.S.[2] Marcelo B. Sztein, M.D.,[1] Rezwanul Wahid, M.B.B.S., Ph.D.,[1] James D. Campbell, M.D., M.S.,[1] Marie-Paule Kieny, Ph.D.,[3] Vasee Moorthy, D.Phil., [3] Egeruan B. Imoukhuede, M.B.B.S.,[4] Tommy Rampling, M.R.C.P.,[4] Francois Roman, M.D.,[5] Iris De Ryck, M.D.,[5] Abbie R. Bellamy, Ph.D., [6] Len Dally, M.S.,[6] Olivier Tshiani Mbaya, M.D.,[7] Aurélie Ploquin, Ph.D.,[7] Yan Zhou, Ph.D.,[7] Daphne A. Stanley, M.S.,[7] Robert Bailer, Ph.D.,[7] Richard A. Koup, M.D.,[7] Mario Roederer, Ph.D.,[7] Julie Ledgerwood, D.O.,[7] Adrian V.S. Hill, D.M.,[4] Ripley Ballou, M.D.[5] Nancy Sullivan, Ph.D.,[7] Barney Graham, M.D.,,[7] Myron M. Levine, M.D.,[1+]

Table of Contents

Ebola Vaccine Trials Team 4

Description of ChAd3-EBO-Z vaccine testing consortium 5

Mali protocol - Inclusion/ Exclusion Criteria 6

U.S. Protocol Inclusion/ Exclusion Criteria 7

Inclusion Criteria 7

Mali Randomization Procedures: 9

Maryland Randomization Procedures: 10

Mali Primary and Secondary Outcome Measures: 11

Maryland Primary and Secondary Endpoints: 12

Mali Sample Size Calculation: 13

Maryland Sample Size Calculation: 14

Supplemental Safety Results 16

Table S1: Laboratory abnormalities observed during 28 days after vaccination with ChAd3-EBO-Z 17

Table S2: Adverse events observed during 28 days after boost with either MVA-BN® Filo (n = 27) or saline (n = 25) of Malian adults who received priming immunization with ChAd3-EBO-Z 18

Table S3: Serological Responses Following a Single Parenteral Administration of ChAd3-EBO-Z of Varying Dosage Levels. 19

Table S4: Serological Responses Following a Single Priming Immunization of ChAd3-EBO-Z at Four Dosage Levels and Following a Booster Dose with MVA-BN®-Filo or with Saline Placebo in N=52 Malian Adults 21

Supplemental Figure 1A: Reverse Cumulative Distributions of ELISA antibody to *Zaire ebolavirus* glycoprotein after administration of varying dosage levels of ChAd3-EBO-Z. 22

Supplemental Figure 1B: Reverse Cumulative Distributions of ELISA antibody to *Zaire ebolavirus* glycoprotein after Boost with MVA-BN®-Filo or placebo (saline) 23

Supplemental Figure 2: Reverse Cumulative Distributions of *Zaire ebolavirus* Mayinga and *Sudan ebolavirus* Gulu glycoprotein ELISA antibodies in plasma of five Malians primed with 1×10^{11} pu of ChAd3-EBO-Z before and after MVA-BN®-Filo boost 24

Supplemental Figure 3: Geometric Mean Titer (GMT) of ELISA antibodies to *Zaire ebolavirus* glycoprotein through 180 days after booster with MVA-BN®-Filo or with saline in Malians primed with a 1×10^{11} pu dose of ChAd3-EBO-Z 25

Supplemental Figure 4: The progressive gating scheme to enumerate antigen specific T cells. 26

Supplemental Figure 5: The proportion of responders (%), magnitude of the memory CD4 and CD8 cytokine (IFN-γ, TNF-α and IL-2) responses stimulated by exposure of T-cells to peptide pools Z1 and Z2 from *Zaire ebolavirus* glycoprotein and pie charts summarizing multifunctionality of the responding memory T-cells. 27

Epilogue - ChAd3-EBO-Z vaccine testingconsortium 29

Ebola Vaccine Trials Team

Mali Team:

Clinical Team – Fadima Cheick Haidara, Fatoumata Diallo, Moussa Doumbia, Flanon Coulibaly, Youssouf Traore, Diakaridia Sidibe, Adama Coulibaly, Ousmane Samaké, Fousseyni Goita, Worokiatou Traoré, Seydou Sissoko, Mamoudou Kodio, Sekou Keita, Kadiatou Diallo, Kaman Dembelé. Oumar Traoré. Oumou Keita, Sogona Faye, Kadiatou Togola, Kindia Kamara, François Diarra, Alhoussein T. Traoré, Oumar Traoré, Glodié Doumbia, Karamoko Deba

Laboratory Team - Awa Traoré, Abdoulaye Sangaré, Seydou Diarra, Abdoul Azize Maiga, Ousmane Diakité

Informatics Team – Uma U. Onwuchekwa, Oualy Diawara, Moussa Traoré, Moussa Fané

Monitoring Team - Rokiatou Dembele, Mamadou B. Diallo, Ballan Sangaré, Ousmane Dembélé

Administrative Support – Araba Maradou, Mossokoro Diallo, Aissata Traore, Aissata Sacko, Alassane Sow, Abdoulaye Coulibaly, Haremakan Diawara, Cheick Oumar Kourouma, Salif Cissé, Mamadou Kourouma, Moussa Sangare

Mali Government – His Excellency Ibrahim Boubacar Keita, the President of the Republic of Mali, Ousmane Koné, Minister of Health, Professor Ousmane Doumbia, Vice-Minister of Health, Dr. Yaya Coulibaly, Director of Mali DPM

U.S. Team:

University of Maryland Clinical team –Myounghee Lee, Wilbur Chen, Nancy Greenberg, Lisa Chrisley, Xiaolin Wang, Melissa Billington, Toni Robinson, Kimberly Wilhelmi, Takiyah Crawford, Panagiota Komninou, Jennifer Courneya

University of Maryland Laboratory Team –Regina Harley, Cathy Storrer

University of Maryland Monitoring Team - Alyson Kwon, Brenda Dorsey, Karen Ball

University of Maryland Support/Administrative – Nicole Eddington-Johnson, Carey Martin, Carol Foreman

Vaccine Research Center, National Institutes of Health Team (VRC 207 Study Team) – Laura Novik, Ingelise Gordon, Adam DeZure, Sandra Sitar, Sarah Plummer, Cynthia S Hendel, LaSonji Holman, Nina Berkowitz, Mary Enama, Galina Yamshchikov, Richard Schwartz, Zonghui Hu, Robert Bailer, Pamela Costner, Floreliz Mendoza, Jamie Saunders, Kathy Zephir, Brenda Larkin, William Whalen, Olga Vasilenko, Iris Pittman, Carmencita Artis, Brandon Wilson, Emily Coates, Christine Nguyen, Pernell Williams, John Gilly, Florence Kaltovich, Michelle Conan-Cibotti, Gretchen Schieber, Judy Stein, Judith Starling, Hope Decederfelt, Phyllis Renehan, Sarah Romano, Meghan Kunchai, Kaitlyn Menard.

Description of ChAd3-EBO-Z vaccine testing consortium

Responding to the precipitous rise in *Zaire ebolavirus* disease in West Africa, in mid-August 2014 the WHO assembled a consortium to accelerate clinical testing of ChAd3-EBO-Z monovalent Ebola vaccine.[1] Single-dose ChAd3-EBO-Z conferred upon NHP 100% protection against challenge with virulent *Zaire ebolavirus* that was lethal for 100% of controls.[2] Consortium institutions included: VRC, NIAID, whose scientists collaborating with Okairos developed ChAd3-EBO-Z and would test clinical specimens by anti-glycoprotein ELISA; GlaxoSmithKline (GSK) Vaccines, to scale-up manufacture; Oxford University, pioneers in clinical testing other ChAd3-vectored vaccines,[3] to initiate clinical trials expeditiously in the UK; CVD-Mali to perform Phase 1b clinical trials in Mali; CVD of the University of Maryland School of Medicine (which, with the Malian Ministry of Health, jointly maintains the CVD-Mali enterprise) to perform trials in U.S. participants; Wellcome Trust to accelerate peer review and funding for the Oxford Phase 1 and Mali Phase 1b trials; WHO, which assembled the consortium and provided coordination.

Mali protocol - Inclusion/ Exclusion Criteria

Inclusion Criteria

The volunteer satisfied all the following criteria to be eligible for the study:

- Healthy adults aged 18 to 50 years
- Able and willing (in the Investigator's opinion) to comply with all study requirements
- Willing to allow the investigators to discuss the volunteer's medical history with his/her health care provider
- For females only, willingness to practice continuous effective contraception during the study and a negative urine pregnancy test on the day(s) of screening and vaccination
- Agreement to refrain from blood donation during the course of the study
- Provide written informed consent

Exclusion Criteria

The volunteer was excluded if one or more of the following conditions applied:

- Participation in another research study involving receipt of an investigational product in the 30 days preceding enrolment, or planned use during the study period
- Prior receipt of an investigational Ebola or Marburg vaccine, a chimpanzee adenovirus vectored vaccine, MVA vectored vaccine or any other investigational vaccine likely to impact on interpretation of the trial data
- Receipt of any live, attenuated vaccine within 28 days prior to enrolment
- Receipt of any subunit or killed vaccine within 14 days prior to enrolment
- Administration of immunoglobulins and/or any blood products within the three months preceding the planned administration of the vaccine candidate
- Any confirmed or suspected immunosuppressive or immunodeficient state, including HIV infection; asplenia; recurrent, severe infections and chronic (more than 14 days) immunosuppressant medication within the past 6 months (inhaled and topical steroids are allowed)
- History of allergic disease or reactions likely to be exacerbated by any component of the vaccine, including urticaria, respiratory difficulty or abdominal pain
- Any history of hereditary angioedema, acquired angioedema, or idiopathic angioedema.
- Any history of anaphylaxis in reaction to vaccination
- Pregnancy, lactation or willingness/intention to become pregnant during the study
- History of cancer (except basal cell carcinoma of the skin and cervical carcinoma in situ)
- History of serious psychiatric condition
- Poorly controlled asthma or thyroid disease
- Seizure in the past 3 years or treatment for seizure disorder in the past 3 years
- Bleeding disorder (e.g., Factor deficiency, coagulopathy or platelet disorder), or prior history of significant bleeding or bruising following IM injections or venipuncture
- Any known history of cardiac disease
- Any other serious chronic illness requiring hospital specialist supervision
- Current anti-tuberculosis prophylaxis or therapy
- Suspected or known current alcohol abuse as defined by an alcohol intake of greater than 42 units every week
- Suspected or known injecting drug abuse in the 5 years preceding enrolment
- Seropositive for hepatitis B surface antigen (HBsAg)
- Travel to an Ebola or Marburg endemic region during the study period or within the previous six months or history of recovery from Ebola or Marburg virus disease.
- Any clinically significant abnormal finding on screening biochemistry or haematology blood tests or urinalysis (see Appendices A & B)
- Any other significant disease, disorder or finding which may significantly increase the risk to the volunteer because of participation in the study, affect the ability of the volunteer to participate in the study or impair interpretation of the study data

U.S. Protocol Inclusion/ Exclusion Criteria

Inclusion Criteria

A volunteer must meet all of the following criteria:

- 18 to 50 years old for Groups 1 and 2; 18 to 65 years old for Groups 3, 4, and 5.
- Available for clinical follow-up through Week 48 after enrollment for Groups 1-4 and through at least Week 4 after enrollment for Group 5 with no planned travel that would preclude completion of the Study Week 4 visit.
- Able to provide proof of identity to the satisfaction of the study clinician completing the enrollment process.
- Able and willing to complete the informed consent process.
- Willing to donate blood for sample storage to be used for future research.
- In good general health without clinically significant medical history.
- Physical examination and laboratory results without clinically significant findings and a body mass index (BMI) ≤ 40 within the 56 days prior to enrollment.
- For Group 3 volunteers only, must have received the VRC-EBODNA023-00-VP (Ebola DNA WT) vaccine in the VRC 206 study.
- ***Laboratory Criteria within 56 days prior to enrollment:***
- Hemoglobin ≥ 11·5 g/dL for women; ≥13·0 g/dL for men.
- White blood cells (WBC) = 3,300-12,000 cells/mm^3.
- WBC differential either within institutional normal range or accompanied by the Principal Investigator (PI) or designee approval.
- Total lymphocyte count ≥ 800 cells/mm^3.
- Platelets = 125,000 – 400,000/mm^3.
- Alanine aminotransferase (ALT) ≤ 1.25 x upper limit of normal.
- Serum creatinine ≤ 1 x upper limit of normal.
- Partial thromboplastin time (PTT) within institutional normal range.
- Prothrombin time (PT) within institutional normal range or accompanied by the Principal Investigator (PI) or designee approval.
- HIV-uninfected as evidenced by a negative FDA-approved HIV diagnostic blood test.
- ***Female-Specific Criteria:***
- Negative β-HCG (human chorionic gonadotropin) pregnancy test (urine or serum) on day of enrollment if woman is presumed to be of reproductive potential.
- Agrees to use an effective means of birth control from at least 21 days prior to enrollment through 24 weeks after study vaccination if presumed to be of reproductive potential.
- **Exclusion Criteria**
- ***A volunteer will be excluded if one or more of the following conditions apply:***
- *Volunteer has received any of the following substances:*
- Investigational Ebola or Marburg vaccine in a prior clinical trial or prior receipt of a cAd3 adenoviral vectored investigational vaccine, except for Group 3 volunteers.
- Immunosuppressive medications within 2 weeks prior to enrollment.
- Blood products within 112 days (16 weeks) prior to enrollment.
- Investigational research agents within 28 days (4 weeks) prior to enrollment.
- Live attenuated vaccines within 28 days (4 weeks) prior to enrollment.
- Subunit or killed vaccines within 14 days (2 weeks) prior to enrollment.
- Current anti-tuberculosis prophylaxis or therapy.

- ***Female-specific criteria:***
- Woman who is breast-feeding or planning to become pregnant during the first 24 weeks after study vaccine administration.
- ***Volunteer has a history of any of the following clinically significant conditions:***
- Serious adverse reactions to vaccines such as anaphylaxis, urticaria (hives), respiratory difficulty, angioedema, or abdominal pain.
- Clinically significant autoimmune disease or immunodeficiency.
- Asthma that is not well controlled.
- Diabetes mellitus (type I or II), with the exception of gestational diabetes.
- Thyroid disease that is not well controlled.
- A history of hereditary angioedema (HAE), acquired angioedema (AAE), or idiopathic forms of angioedema.
- Idiopathic urticaria within the last 1 year.
- Hypertension that is not well controlled.
- Bleeding disorder diagnosed by a doctor (e.g. factor deficiency, coagulopathy, or platelet disorder requiring special precautions) or significant bruising or bleeding difficulties with IM injections or blood draws.
- Malignancy that is active or history of a malignancy that is likely to recur during the period of the study.
- Seizure in the past 3 years or treatment for seizure disorder in the past 3 years.
- Asplenia or functional asplenia.
- Psychiatric condition that precludes compliance with the protocol; past or present psychoses; or within five years prior to enrollment, history of a suicide plan or attempt.
- Any medical, psychiatric, social condition, occupational reason or other responsibility that, in the judgment of the investigator, is a contraindication to protocol participation or impairs a volunteer’s ability to give informed consent.

Mali Randomization Procedures:

Mali Trial Site:

A Phase 1b open label vaccine trial was initiated at CVD-Mali, Bamako, Mali in healthy adults 18-50 years of age who were to be vaccinated shortly after the first 5 vaccinees in Oxford had been immunized. Volunteers were provided background on the trial and informed consent obtained prior to enrollment. Because of the limited number of investigational vaccine doses available, the Malian Phase 1b trial was initially designed to test only two dosage levels, 2.5×10^{10} viral particle units (pu) and 5.0×10^{10} pu, in an open-label trial. When extra doses became available, following approval of the modified protocol additional participants were randomly allocated to receive 2.5×10^{10} or 5.0×10^{10} pu in double-blind fashion and smaller groups were given 1×10^{10} or 1×10^{11} pu.

The study statistician generated randomization sequences for randomized segments of the Mali trial for groups 3B-3C using blocked randomization (block size 6) and for the booster using simple randomization. Upon enrollment, each subject was assigned a randomization number from the electronic data entry system that corresponded to a treatment on a randomization list available only to the un-blinded study pharmacist and vaccine administrator.

Maryland Randomization Procedures:

Maryland Trial Site:

Randomization was accomplished through an on-line database and randomization software (AdvantageEDC) managed by the EMMES Corporation (Rockville, MD). To complete the enrollment process, the clinician completed the Eligibility Checklist electronic case report form (eCRF). Upon successful enrollment, a protocol and site-specific subject identification number was assigned. Each subject received a unique study identification number. After an enrollment was completed, AdvantageEDC displayed the study subject ID and the Treatment Assignment (high does or low dose) on the Enrollment Confirmation screen.

The Enrollment Confirmation screen was printed and maintained in the subject's file. The data displayed on the screen was accessible after enrollment on the Enrollment Confirmation screen in AdvantageEDC.

In the event that AdvantageEDC was not accessible to the site at the desired time of enrollment, investigators were instructed to contact the CPSC EMMES Project Manager to initiate backup manual enrollment procedures.

Mali Primary and Secondary Outcome Measures:

Primary Outcome Measures

The specific endpoints for safety and reactogenicity will be actively (solicited) and passively (unsolicited) collected data on adverse events.
The following parameters will be assessed for all study groups:

- Occurrence of solicited local reactogenicity signs and symptoms for 7 days following the vaccination
- Occurrence of solicited systemic reactogenicity signs and symptoms for 7 days following the vaccination
- Occurrence of unsolicited adverse events for 28 days following the vaccination
- Change from baseline for safety laboratory measures
- Occurrence of serious adverse events through last study visit

Secondary Outcome Measures

Ebolavirus specific immunogenicity will be assessed by a variety of immunological assays.

The primary immunogenicity outcome measures are ELISA and neutralization antigen-specific assays for antibody responses and intracellular cytokine staining (ICS) assay for T cell responses.

Exploratory outcome measures will include ex-vivo ELISPOT, plasma blast assays and flow cytometry performed with research samples collected at study timepoints as well as other immunogenicity assays throughout the study and evaluation of genetic factors associated with immune responses may be completed as exploratory evaluations. Vaccine-induced mRNA expression profiles during 1 week after vaccination may also be performed as an exploratory evaluation.

Both primary and exploratory immunology may involve collaboration with other specialist laboratories, including laboratories outside of Mali (including UK, Europe and USA). This would involve transfer of serum/plasma and/or peripheral blood mononuclear cells (PBMC), but samples would be de-identified. Volunteers will be consented for this during the process of obtaining informed consent for the study.

De-identified clinical specimens consisting of serum, plasma, and PBMCs from Malian subjects will be sent to the VRC, NIH, which will serve as the immune response measurement reference laboratory for the Mali and Oxford Phase I trials. In this way, standardized assays will be used to measure immune responses among subjects from all three field sites.

Maryland Primary and Secondary Endpoints:

Endpoints

Primary Endpoints: Safety

Assessment of product safety will include clinical observation and monitoring of clinical chemistry and hematology parameters. Safety will be closely monitored after injection and evaluated through 48 weeks after the study injection. See VRC protocol 207 for details and specified time points. The following parameters will be assessed for all study groups:

- Occurrence of solicited local reactogenicity signs and symptoms for 7 days following the vaccination
- Occurrence of solicited systemic reactogenicity signs and symptoms for 7 days following the vaccination
- Change from baseline for safety laboratory measures
- Occurrence of adverse events of all severities through 48 weeks after the injection
- Occurrence of serious adverse events through last study visit

Secondary Endpoints: Immunogenicity

The primary immunogenicity endpoints are ELISA and neutralization antigen-specific assays for antibody responses and intracellular cytokine staining (ICS) assay for T cell responses. The principal time point for antibody and T cell responses is Week 4 after vaccination.

Mali Sample Size Calculation:

Sample size calculation

This study was designed as an observational and descriptive safety study, where 91 volunteers were vaccinated with a single dose of ChAd3-EBO-Z at either 1.0 x 10^{10} vp (N=10), 2·5 x 10^{10} vp (N=35), 5·0 x 10^{10} vp (N=35) or 1·0 x 10^{11} vp (N=11). The design was based, in part, upon the availability of vaccine doses. To that end, the Malian trial was initially designed to test 2·5x10^{10} and 5·0x10^{10} pu in an open-label trial. When extra doses became available, additional participants were randomly allocated to receive 2·5x10^{10} or 5·0x10^{10} pu in double-blind fashion; smaller groups were given 1x10^{10} or 1x10^{11} pu. Similarly, based on the available doses of MVA-BN® Filo, 56 participants were randomized to receive either active booster or saline placebo.

The sample size for this phase 1b trial balanced the need to avoid exposing a large group to an unknown risk with the need to obtain preliminary estimates of immunogenicity and safety in a time-critical manner from an adequate sample. Group sample sizes were based on availability of study product. This sample size should have allowed determination of the magnitude of the outcome measures, especially of serious and severe adverse events, rather than aiming to obtain statistical significance for differences between groups.

Maryland Sample Size Calculation:

Sample Size and Accrual

For Part 1, the study design was Phase 1 dose escalation based on a target accrual of 20 healthy adult participants divided equally among 2 dose groups. After the initial safety review, in Part 2, a Phase 1b evaluation of safety and immunogenicity continued by evaluation of the Zaire component of the vaccine in about 20 subjects who received the ChAd3-EBO-Z vaccine.

Power Calculations for Safety

The goal of the safety evaluation for this study was to identify safety concerns associated with vaccination. Primary sample size calculations for safety were expressed in terms of the ability to detect serious adverse experiences.

The ability of the study to identify SAEs was expressed in terms of the probability of observing a certain number of serious adverse events. Useful values were the minimum true rate such that the probability of observing at least one event was at least 90%, and the maximum true rate such that the probability of not observing any event was at least 90%. Within each group (n=10), there was over 90% chance to observe at least 1 SAE if the true rate was at least 0·206 and over 90% chance to observe no SAE if the true rate was no more than 0·01.

Probabilities of observing 0 or more than 1 serious adverse event within each group are presented below for a range of possible true event rates. These calculations provided a more complete picture of the sensitivity of this study design to identify potential safety problems with the vaccine.

Probability of Events for Different Safety and Immunogenicity Scenarios Within a Group (n=10**)**

True Event rate	Pr (observing 0 event)	Pr (observing more than 1 event)
0·005	0·951	0·001
0·010	0·904	0·004
0·020	0·817	0·016
0·035	0·700	0·046
0·050	0·599	0·086
0·100	0·349	0·264
0·150	0·197	0·456
0·200	0·107	0·624
0·300	0·028	0·851

The following table gives the upper and lower bounds for 95% exact binomial confidence intervals of the true SAE rate at all possible numbers of events within each group. For a group with n=10 vaccinees, if none experience SAE, the 95% exact confidence interval has upper bound 0·308.

95% Confidence Intervals for the True Rate at All Possible Observed Rates Within a Group (n=10)

	**95% Confidence Interval**	
**Observed Rate**	**Lower Bound**	**Upper Bound**
0/10	0	0·308
1/10	0·003	0·445
2/10	0·025	0·556
3/10	0·067	0·652
4/10	0·122	0·738
5/10	0·187	0·813
6/10	0·262	0·878
7/10	0·348	0·933
8/10	0·444	0·975
9/10	0·555	0·997
10/10	0·692	1

The calculations are also applicable to the immunogenic response rates, and give the exact 95% confidence interval of the true response rate over possible number of responses out of the 10 subjects. For example, if we observed 5 responses among the 10 vaccinees, the 95% exact binomial confidence interval of the true response rate would range from 0·187 to 0·813.

Supplemental Safety Results

Post priming dose

Most AEs in recipients of all dosage levels were mild, with no suspected unexpected serious adverse reactions (SUSARs). There was one serious adverse event (SAE) in Mali (tuberculous peritonitis), unrelated to vaccine. The predominant objective systemic adverse reaction was fever, which occurred in 7 of 91 Malian and 4 of 20 US participants. Of Malians with fever, five were recipients of $5x10^{10}$ pu and two of $1x10^{11}$ pu. All four US participants with documented fever received $1x10^{11}$ pu. Of 11 fevers, 10 resolved by 24 hours after vaccination. One fever of 37·6°C occurred in a Malian volunteer on day 2 after vaccination. Fever occurring in one Malian ($5x10^{10}$ pu) and two US vaccinees ($1x10^{11}$ pu) exceeded 38·5° C and was accompanied by systemic symptoms such as fatigue, myalgia, arthralgia, headache, chills or nausea. No febrile response persisted for more than 24 hours.

Within the first 7 days after vaccination of Malian participants, the most frequently observed laboratory abnormality was lymphopenia. Most episodes (11/12) were noted on Day 1 post-vaccination and lasted one week. Other hematological abnormalities observed among recipients of the $2{\cdot}5x10^{10}$ or $5{\cdot}0x10^{10}$ pu dosage included thromobocytopenia (2/70), neutropenia (6/70), and anemia (4/70). Additionally, there was an episode of mildly elevated alanine aminotransferase (ALT) that resolved within 6 days. No hematological abnormalities were observed in the $1x10^{10}$ pu dosage group over the first 7 days of follow up. There was also one participant who had received the $2{\cdot}5x10^{11}$ pu dose and had mild numbness at the injection site and mild vomiting on the day of vaccination. Over the remaining 28-day follow up period, the following events occurred and were possibly related to study vaccination: 1 episode of moderate lymphopenia that resolved in 23 days, 3 episodes each of mild neutropenia and anemia that lasted 14 to 16 days. These observations are summarized in Table S1.

Among U.S. participants who received the $1x10^{10}$ pu dosage, 1 mild aPTT prolongation (Day 14), and 3 mild leukopenias on Day 3 with 1 concomitant mild neutropenia were observed. U.S. recipients of $1x10^{11}$ pu exhibited 3 mild aPTT prolongations (on Day 14 x 2, Day 28 x 1). These events are summarized in Table S1. All of these events resolved spontaneously.

Post booster (Table S2)

Adverse reactions were uncommon among the 27 Malian participants boosted with MVA-BN Filo. Over the first 7 days, the most common local reaction was pain at the injection site; 13 of the episodes were mild and 2 were moderate in intensity. Additionally 3 participants complained of numbness in the area of the injection site. One participant had a temperature of 37·7 °C on the day after vaccination which was associated with moderate pain at the injection site and mild myalgia, headache and fatigue. Another participant had a temperature of 38·0°C on day 1 and associated with moderate pain at the injection site and mild myalgia, headache and fatigue. A thick smear for malaria parasites was negative. Both were well by the third day after vaccination.

Hematological abnormalities within the first 7 days after vaccination included: 1 episode of moderate anemia that lasted 1 week, 2 episodes of mild and 1 moderate lymphopenia that lasted 1 to 3 days and 1 episode of mild neutropenia that lasted 1 week. Another participant also experienced a mild elevation of ALT that began on day 2 and lasted 5 days. Other adverse events within the first 7 days after vaccination included two episodes of mild cough and single episodes of diarrhea, tinnitus and conjunctivitis. These events lasted 2 to 8 days. There were also episodes of mild cough and elevated ALT that occurred on day 8 and 14, respectively.

Of the 25 Malian participants allocated to a saline booster, one participant had an isolated temperature of 37·9°C recorded the 5th day after vaccination. Hematological abnormalities observed within 7 days included 4 cases of mild neutropenia and mild leukopenia that lasted from 3 days to almost 8 weeks. There was also 1 episode of elevated ALT that began on day 7 and lasted 1 week.

Table S1: Laboratory abnormalities observed during 28 days after vaccination with ChAd3-EBO-Z

Event	Dosage group	Number of episodes observed	Number of episodes observed in first 7 days of follow up*	Mean days to resolution	Range of days to resolution**	Grade
**Mali Study**						
Anemia	2·5 x 10^{10} p.u.	4	1	12	6 – 16	Mild
	5 x 10^{10} p.u.	3	3	18·3	6 – 36	Mild
Neutropenia	1·0 x 10^{10} p.u.	1	0	-	14	Mild
	2·5 x 10^{10} p.u.	2	1	10	6 – 14	Mild
		1	1	-	29	Moderate
	5 x 10^{10} p.u.	5	4	15	6 – 42	Mild
Lymphopenia	2·5 x 10^{10} p.u.	4	4	5·25	4 – 6	Mild
		2	1	14·5	6 – 23	Moderate
	5 x 10^{10} p.u.	3	3	6	6	Mild
	1 x 10^{11} p.u.	3	3	6	6	Mild
		1	1	-	6	Moderate
Thrombocytopenia	2·5 x 10^{10} p.u.	1	1	-	6	Severe
	5 x 10^{10} p.u.	1	1	-	6	Moderate
Elevated ALT[&]	2·5 x 10^{10} p.u.	1	1	-	6	Mild
**U. S. Study**						
Prolonged PTT	1 x 10^{10} p.u.	1	0	-	98	Mild
	1 x 10^{11} p.u.	3	0	62·5	14-139	Mild
Leukopenia	1 x 10^{10} p.u.	2	2	19·5	11-28	Mild
Neutropenia	1 x 10^{10} p.u.	1	1	-	165	Mild
Lymphopenia	1 x 10^{10} p.u.	3	3	63·3**	11-165	Mild

* This represents a subset of those observed in the column to the left.
**Days to resolution skewed by intervals between visits and participant availability
[&] ALT = Alanine aminotransferase

Table S2: Adverse events observed during 28 days after boost with either MVA-BN® Filo (n = 27) or saline (n = 25) of Malian adults who received priming immunization with ChAd3-EBO-Z

Event	Product received	Number of episodes observed in first 7 days of follow up	Number of episodes observed from days 8-28	Mean days to resolution	Range of days to resolution*	Grade
Anemia	MVA-BN® Filo	1	0	-	2	Moderate
Neutropenia	MVA-BN® Filo	1	0	-	7	Mild
	Saline	4	1	13·8	5 - 27	Mild
Lymphopenia	MVA-BN® Filo	2	0	2	1 - 3	Mild
	MVA-BN® Filo	1	0	-	2	Moderate
Elevated ALT[&]	MVA-BN® Filo	1	1	9·5	5 - 14	Mild
	Saline	1	0	-	7	Mild
Cough	MVA-BN® Filo	2	1	6·7	5 - 8	Mild
Conjunctivitis	MVA-BN® Filo	1	0	-	3	Mild
Diarrhea	MVA-BN® Filo	1	0	-	2	Mild
Tinnitus	MVA-BN® Filo	1	0	-	8	Mild

*Days to resolution skewed by intervals between visits and participant availability
[&] ALT = Alanine aminotransferase

Table S3: Serological Responses Following a Single Parenteral Administration of ChAd3-EBO-Z of Varying Dosage Levels.

	**Malian Adults**				**Maryland Adults**	
	**Vaccine Dosage Level**				**Vaccine Dosage Level**	
	$1x10^{10}$ pu* (N=10)	$2.5x10^{10}$ pu (N=34)	$5x10^{10}$ pu (N=35)	$1x10^{11}$ pu (N=11)	$1x10^{10}$ pu (N=10)	$1x10^{11}$ pu (N=10)
Positive Response by Day 14 n/N [%] (95% CI)	NA	4/5 [80·0] (28·4 - 99·5)	15/20 [75·0] (50·9 - 91·3)	7/11 [63·6] (30·8 - 89·1)	NA	NA
Positive Response by Day 28	10/10 [100] (69·2 - 100)	33/34 [97·1] (84·7 - 99.9)	29/35 [82·9] (66·4 - 93·4)	11/11 [100] (71·5 - 100)	10/10 [100] (69·2 - 100)	10/10 [100] (69·2 - 100)
Geometric Mean Titer, Day 14 (95% CI)	N/A	97·1 [5] (29·6 - 318·0)	270·8 [20] (119·4 - 613·8)	116.2 [11] (37·1 - 363·9)	NA	NA
Geometric Mean Titer, Day 28 (95% CI)	295·0 (114·8-758·2)	220·4 (155·9-311·6)	466·0 (289·1-750·9)	1446·9 (759·4-2756·8)	531·5 (249·4 - 1132·5)	1255·9 (379.7 - 4154·2)
Day 28 Reciprocal Titers ≥ 500† n/N [%] (95% CI)	3/10 [30·0] (6·7-65·2)	5/34 [14·7] (5·0- 31·1)	15/35 [42·9] (26·3-60·6)	10/11 [90·9] ‖ (58·7-99·8)	5/10 [50·0] (18·7 - 81·3)	7/10 [70·0] (34·8 - 93·3)
Day 28 Reciprocal Titers ≥ 1000‡ n/N [%]	1/10 [10] (0·3-44·5)	3/34 [8·8] (1·9-23·7)	7/35 [20·0] (8·4-36·9)	10/11 [90·9]¶ (58·7-99·8)	3/10 [30·0] (6·7-65·2)	6/10 [60·0] (26·2-87·8)
Day 28 Reciprocal Titers ≥ 1500∫	1/10 [10] (0·3-44·5)	2/34 [5·9] (0·7-19·7)	6/35 [17·1] (6·-33·6)	6/11 [54·5]** (23·4-83·3	3/10 [30·0] (6·7 - 65·2)	6/10 [60·0] (26·2 - 87·8)
Baseline ChAd3 Neutralizing Antibody Reciprocal Titer ≥ 200	2/10 [20·0]†† (2·5-55·6)	4/34 [11·8]†† (3·3-27·5)	3/35 [8·6]†† (1·8-23·1)	0/11 [0]†† (0-28·5)	-	-
Baseline Ad5 Neutralizing Antibody Reciprocal Titer ≥ 200	6/10 [60·0] (26·2-87·8)	30/34 [88·2] (72·6-96·7)	30/35 [85·7] (69·7-95·2)	8/11 [72·7] (39·0-94·0)	-	-

* pu = viral particle units

Among non-human primates vaccinated with Adenovirus 5-vectored candidate Ebola vaccines that elicit anti-glycoprotein antibodies and were challenged ~ one month later with virulent Ebolavirus that resulted in 100% mortality among unvaccinated controls:[4]

† Vaccinated animals that mounted a reciprocal titer ≥ 500, exhibited 74·4% vaccine efficacy against fatal Ebolavirus illness.

‡ Vaccinated animals that mounted a reciprocal titer ≥ 1000 exhibited 77·1% vaccine efficacy against fatal Ebolavirus illness.

∫ Vaccinated animals that mounted a reciprocal titer ≥ 1500 exhibited 84·6% vaccine efficacy against fatal Ebolavirus illness.

Among non-human primates vaccinated with ChAd3-vectored candidate Ebola vaccines that elicit anti-glycoprotein antibodies and were challenged ~ one month later with virulent Ebolavirus that resulted in 100% mortality among unvaccinated controls, vaccinated animals that mounted a reciprocal titer ≥ 976 exhibited 100% vaccine efficacy against fatal Ebolavirus illness.[2]

‖ The proportion of subjects who reached a reciprocal titer $\geq$ 500 among recipients of $1x10^{11}$ pu was significantly higher than the proportion who reached this titer after vaccination with $5x10^{10}$ pu (p=0·0062), $2.5x10^{10}$ pu (p<0·0001) or $1x10^{10}$ pu (p=0·0075).

¶ The proportion of subjects who reached a reciprocal titer $\geq$ 1000 among recipients of $1x10^{11}$ pu was significantly higher than the proportion who reached this titer after vaccination with $5x10^{10}$ pu (p<0·0001), $2.5x10^{10}$ pu (p<0·0001) or $1x10^{10}$ pu (p=0·00035).

** The proportion of subjects who reached a reciprocal titer $\geq$ 1500 among recipients of $1x10^{11}$ pu was significantly higher than the proportion who reached this titer after vaccination with $5x10^{10}$ pu (p=0·0223), $2.5x10^{10}$ pu (p=0·0013) or $1x10^{10}$ pu (p=0·063).

†† There were no significant differences in the prevalence of ChAd3 neutralizing antibody titers among the different vaccine dosage groups at baseline (P>0·05 for all group-wise comparisons). Some reports have described diminished or modified immune responses to foreign antigens in recipients of Ad5-vectored vaccines who had elevated baseline anti-Ad5 neutralizing antibodies,[5] and in developing countries Ad5 antibodies are highly prevalent.[6-8] Anti-Ad5 antibodies were common in the Malians participants in this study, while anti-ChAd3 antibodies were infrequent (the difference in prevalence being significant [P<0·001] for all groups except the $1x10^{10}$ pu group). The median baseline anti-ChAd3 titer in the 91 Malian participants, 18 (95% CI, 4-45), was significantly lower than the median baseline anti-Ad5 1379 (872-2162) titers (P<0·0001, Wilcoxon, 2-sample test). High-titer ChAd3 neutralizing antibodies have also been found to be uncommon elsewhere in Africa and in other developing countries.[8]

Table S4: Serological Responses Following a Single Priming Immunization of ChAd3-EBO-Z at Four Dosage Levels and Following a Booster Dose with MVA-BN®-Filo or with Saline Placebo in N=52 Malian Adults

	**Response of Malians Following Priming Vaccination**						**Response of Malians Following Booster (any priming dosage)**	
	$1x10^{10}$ PU (N=10)	$2.5x10^{10}$ PU (N=13)	$5x10^{10}$ PU (N=19)	$1x10^{11}$ PU (N=10)	All four dosage levels (N=52)		$1x10^{8}$ PFU of MVA-BN®-Filo (N=27)	Saline placebo (N=25)
Positive Response by Day 7 n/N [%] (95% CI)	2/10 [20·0] (2·5-55·6)	3/13 [23·1] (5·0-53·8)	2/19 [10·5] (1·3-33·1)	0/10 [0] (0-30·8)	7/52 [13·5] (5·6 – 25·8)		27/27 [100] (87·2-100)	21/24 [87·5] (67·6 – 97·3)
Positive Response by Day 28	10/10 [100] (69·2-100)	13/13 [100] (75·3 – 100)	18/19 [94·7] (74·0 – 99·9)	10/10 [100] (69·2-100)	51/52 [98·1] (89·7 - >99·9)		27/27 [100] (87·2-100)	20/24 [83·3] (62·6 – 95·3)
Geometric Mean Titer [N], Day 7 (95% CI)	16·2 [10] (8·2 - 32·2)	6·4 [13] (3·0 - 13·6)	4·7 [19] (1·7 - 13·2)	6·5 [10] (1·9 - 21·7)	-		11209·3 [27] (8552·6 – 14691·4)	341·3 [24] (182·2 – 637·1)
Geometric Mean Titer [N], Day 28	295·0 [10] (114·8-758·2)	204·6 [13] (99·9 – 423·5)	555·8 [19] (282·2 – 1094·6)	1493·6 (727·6–3065·9)	-		9279·6 [27] (7193·2 – 11971·2)	261·3 [24] (173·9 – 392·7)
Day 7 Reciprocal Titers ≥ 500*	0/10 [0] (0 - 30·8)	0/13 [0] (0 - 24·7)	1/19 [5·3] (0·1 - 26·0)	0/10 [0] (0 - 30·8)	1/52 [1·9] (<0·1 – 10·2)		27/27 [100] (87·2 – 100)	7/24 [29.2] (12·6 – 51·0)
Day 7 Reciprocal Titers ≥ 1000	0/10 [0] (0 - 30·8)	0/13 [0] (0 - 24·7)	1/19 [5·3] (0·1 - 26·0)	0/10 [0] (0 - 30·8)	1/52 [1·9] (<0·1 – 10·2)		27/27 [100] (87·2 – 100)	5/24 [20·1] (7·1 – 42·2)
Day 28 Reciprocal Titers ≥ 500*	3/10 [30·0] (1·6 – 58·4)	2/13 [15·4] (1·9 – 45·4)	8/19 [42·1] (20·6 – 66·5)	9/10 [90·0] (55·5 – 99·7) ∫	22/52 [42·3] (28·7 – 56·8)		27/27 [100] (87·2 – 100)	6/24 [25·0] (9·8 – 46·7)
Day 28 Reciprocal Titers ≥ 1000†	1/10 [10] (0·2-4·5)	2/13 [15·4] (1·9 – 45·4)	3/19 [15·8] (3·4 – 39·6)	9/10 [90·0] (55·5 – 99·7)‖	15/52 [28·8] (17·1 – 43·1)		27/27 [100] (87·2 – 100)	3/24 [12·5] (2·7 – 32·4)
Day 28 Reciprocal Titers ≥ 1500‡	1/10 [10] (0·2-4·5)	1/13 [7·7] (0·2 – 36·0)	3/19 [15.8] (3·4 – 39·6)	6/10 [60·0] (26·2 – 87·8)¶	11/52 [21·2] (11·1 – 34·7)		27/27 [100] (87·2 – 100)	0/24 [0] (0 – 14·2)

Among non-human primates vaccinated with candidate non-replicating adenovirus-vectored Ebola vaccines that elicit anti-glycoprotein antibodies and were subsequently challenged ~ one month later with virulent Ebolavirus that resulted in 100% mortality among unvaccinated controls:[4]

* Vaccinated animals that mounted a reciprocal titer ≥ 500, exhibited 74·4% vaccine efficacy against fatal Ebolavirus illness.

† Vaccinated animals that mounted a reciprocal titer ≥ 1000 exhibited 77·1% vaccine efficacy against fatal Ebolavirus illness.

‡ Vaccinated animals that mounted a reciprocal titer ≥ 1500 exhibited 84·6% vaccine efficacy against fatal Ebolavirus illness.

Among non-human primates vaccinated with ChAd3-vectored candidate Ebola vaccines that elicit anti-glycoprotein antibodies and were challenged ~ one month later with virulent Ebolavirus that resulted in 100% mortality among unvaccinated controls, vaccinated animals that mounted a reciprocal titer ≥ 976 exhibited 100% vaccine efficacy against fatal Ebolavirus illness.[2]

∫ The proportion of boosted subjects who reached a reciprocal titer ≥ 500 among recipients of $1x10^{11}$ pu was significantly higher than the proportion who reached this titer after vaccination with $5x10^{10}$ pu (p=0·019), $2.5x10^{10}$ pu (p<0·0001) or $1x10^{10}$ pu (p=0·0198)

‖ The proportion of boosted subjects who reached a reciprocal titer ≥ 1000 among recipients of $1x10^{11}$ pu was significantly higher than the proportion who reached this titer after vaccination with $5x10^{10}$ pu (p=0·0002), $2.5x10^{10}$ pu (p<0·0001) or $1x10^{10}$ pu (p=0·0011)

¶ The proportion of boosted subjects who reached a reciprocal titer ≥ 1500 among recipients of $1x10^{11}$ pu was significantly higher than the proportion who reached this titer after vaccination with $5x10^{10}$ pu (p=0.0317), $2.5x10^{10}$ pu (p=0·0186) or $1x10^{10}$ pu (p=0·0573)

Note: Among the 27 participants boosted with MVA-BN®-Filo, 5 were primed with $1x10^{10}$ pu of ChAd3-EBO-Z, 7 with $2·5x10^{10}$ pu, 10 with $5x10^{10}$ pu and 5 with $1x10^{11}$ pu.

Among the 25 participants boosted with saline placebo, 5 were primed with $1x10^{10}$ pu of ChAd3-EBO-Z, 6 with $2·5x10^{10}$ pu, 9 with $5x10^{10}$ pu and 5 with $1x10^{11}$ pu.

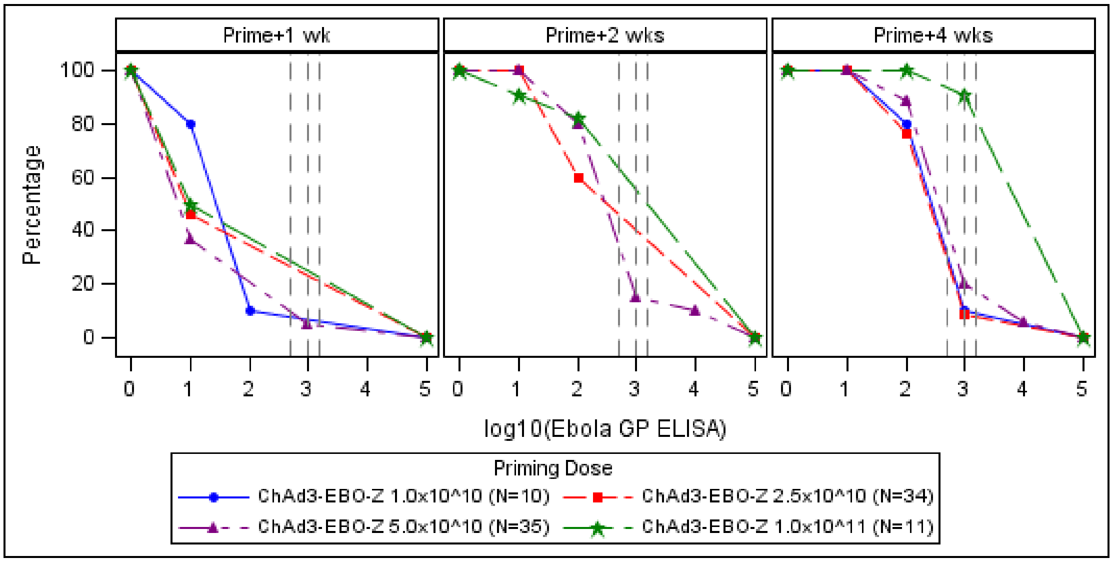

Supplemental Figure 1A: Reverse Cumulative Distributions of ELISA antibody to *Zaire ebolavirus* glycoprotein after administration of varying dosage levels of ChAd3-EBO-Z

Plots display the percentage of subjects with ELISA reciprocal titers of anti-glycoprotein antibody of at least the value indicated on the x-axis (log-10 scale), by ChAd3-EBO-Z priming dosage level. Three post-prime visits are displayed in separate panels.

Reference lines are plotted at ELISA titers of 500, 1000, and 1500 because non-human primates vaccinated with non-replicating adenovirus vectors expressing *Ebolavirus* glycoproteins and that achieved these reciprocal titers exhibited 74.4%, 77.1%, and 84.6%, vaccine efficacy respectively, against fatal illness upon challenge with wild type *Ebolavirus*.

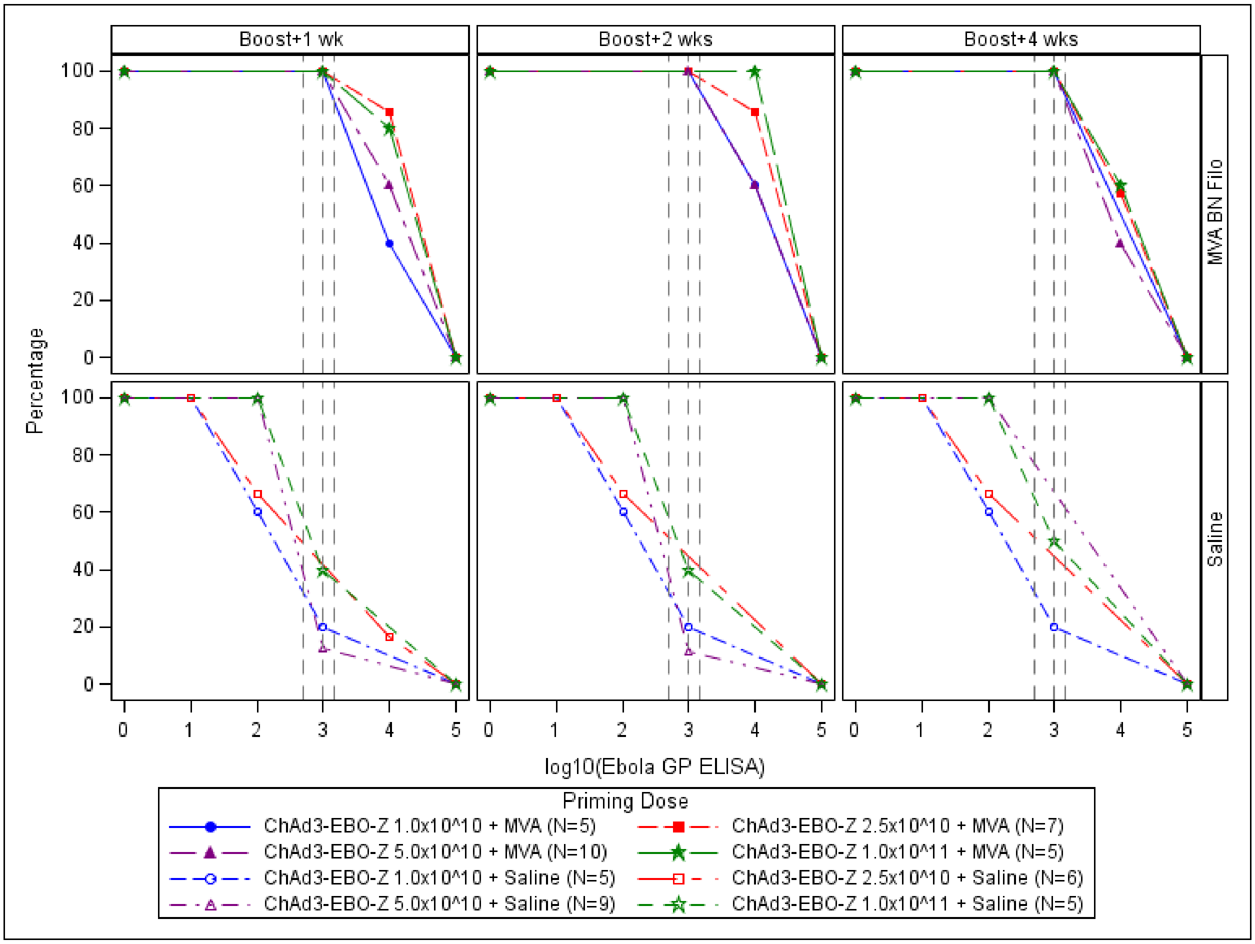

Supplemental Figure 1B: Reverse Cumulative Distributions of ELISA antibody to *Zaire ebolavirus* glycoprotein after Boost with MVA-BN®-Filo or placebo (saline)

Plots display the percentage of subjects with ELISA anti-glycoprotein antibody reciprocal titers of at least the value indicated on the x-axis (log-10 scale) following priming with ChAd3-EBO-Z at different dosage levels. Three post-boost visits are displayed in separate panels for subjects who received MVA boost (top row) or saline placebo (bottom row). Reference lines are plotted at reciprocal ELISA titers of 500, 1000, and 1500

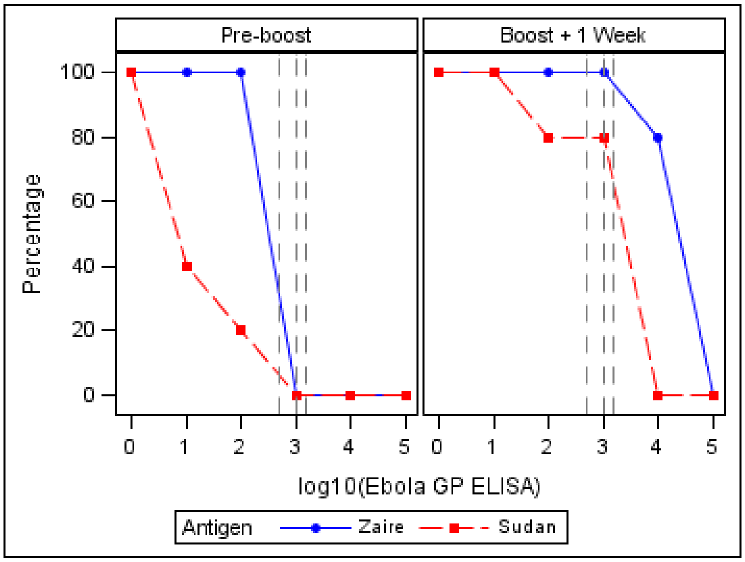

Supplemental Figure 2: Reverse Cumulative Distributions of *Zaire ebolavirus* Mayinga and *Sudan ebolavirus* Gulu glycoprotein ELISA antibodies in plasma of five Malians primed with $1x10^{11}$ pu of ChAd3-EBO-Z before and after MVA-BN®-Filo boost

Plots display the percentage of subjects with ELISA titers of at least the value indicated on the x-axis (log-10 scale) by Antigen pre-boost (left panel) and 1 week post-boost (right panel). Reference lines are plotted at ELISA titers of 500, 1000, and 1500 because non-human primates vaccinated with non-replicating adenovirus vectors expressing *Ebolavirus* glycoproteins and that achieved these reciprocal titers exhibited 74.4%, 77.1%, and 84.6%, vaccine efficacy respectively, against fatal illness upon challenge with wild type *Ebolavirus*.

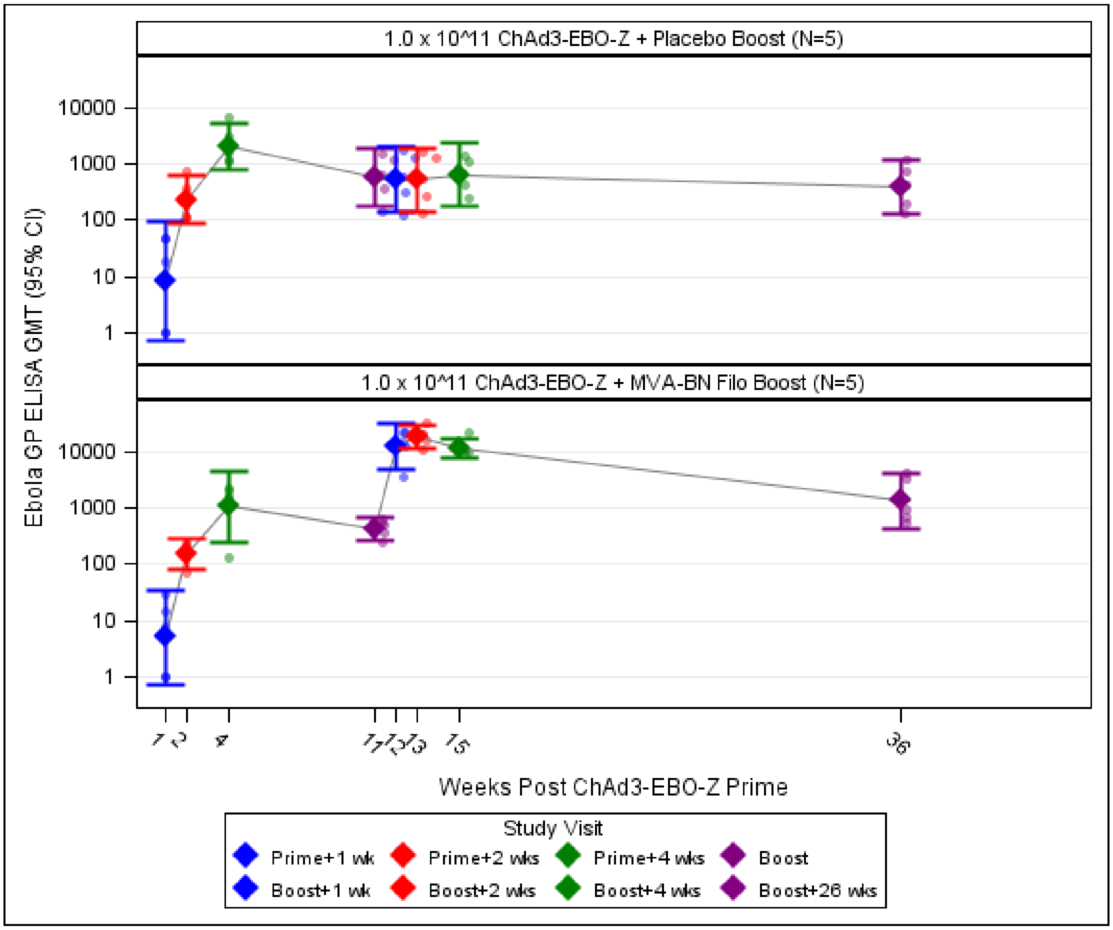

Supplemental Figure 3: Geometric Mean Titer (GMT) of ELISA antibodies to *Zaire ebolavirus* glycoprotein through 180 days after booster with MVA-BN®-Filo or with saline in Malians primed with a $1x10^{11}$ pu dose of ChAd3-EBO-Z

By Day 180 after the boost, the GMT of anti-glycoprotein antibodies for subjects who received the MVA was 1,365 (95%CI 433-4,297) compared with 391 (95%CI 125-1,224) for those who received placebo (Wilcoxon test, p = 0.14). Nevertheless, in the saline-boosted participants there was only a modest decline of antibodies over the many weeks following peak response at 4 weeks post-prime.

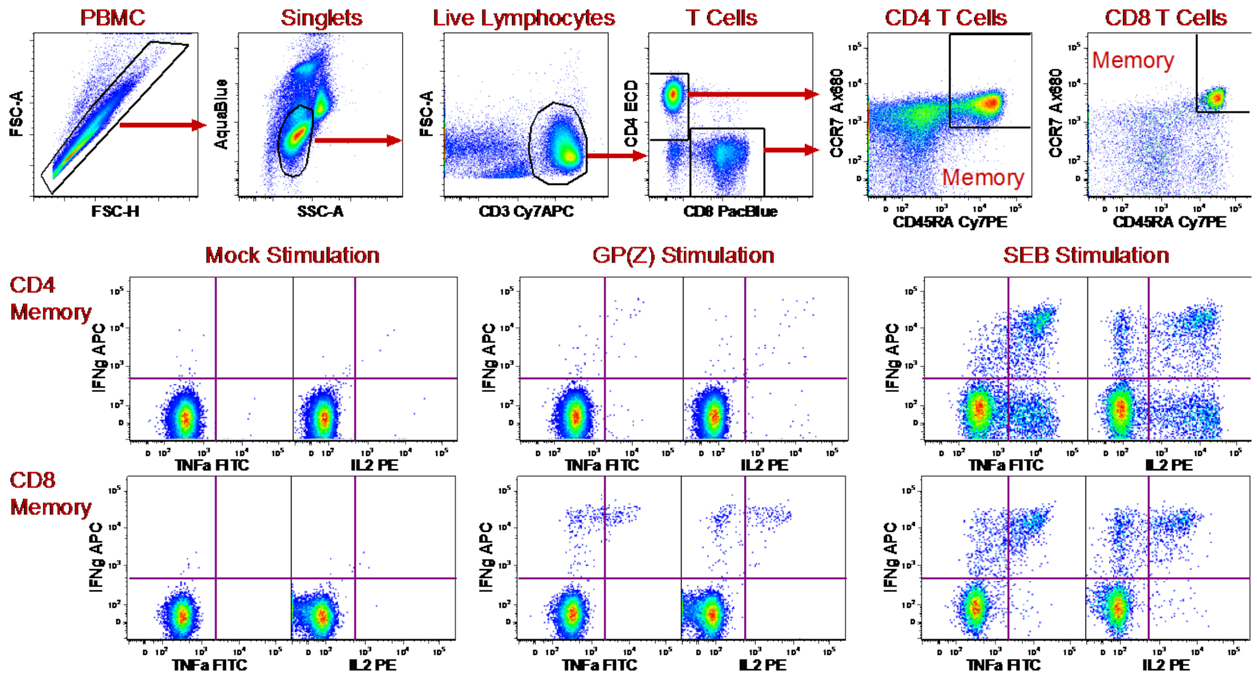

Supplemental Figure 4: The progressive gating scheme to enumerate antigen specific T cells.

The top row shows gating to identify, in succession, single cells, live lymphocytes, T-cells, and CD4 or CD8 T-cell lineages. Within the lineages, memory T-cells are defined as those that are negative for expression of either CCR7 (CD197) or CD45RA. The bottom rows depict PBMC from a representative vaccinated participant following mock stimulation, peptide stimulation, or the positive control (*Staphylococcal* enterotoxin B (SEB)) stimulation. The fraction of TNF, IL-2, or IFN-γ expressing cells was defined based on individual gates; Boolean gate combinations were used to enumerate combinations of expression of cytokines.

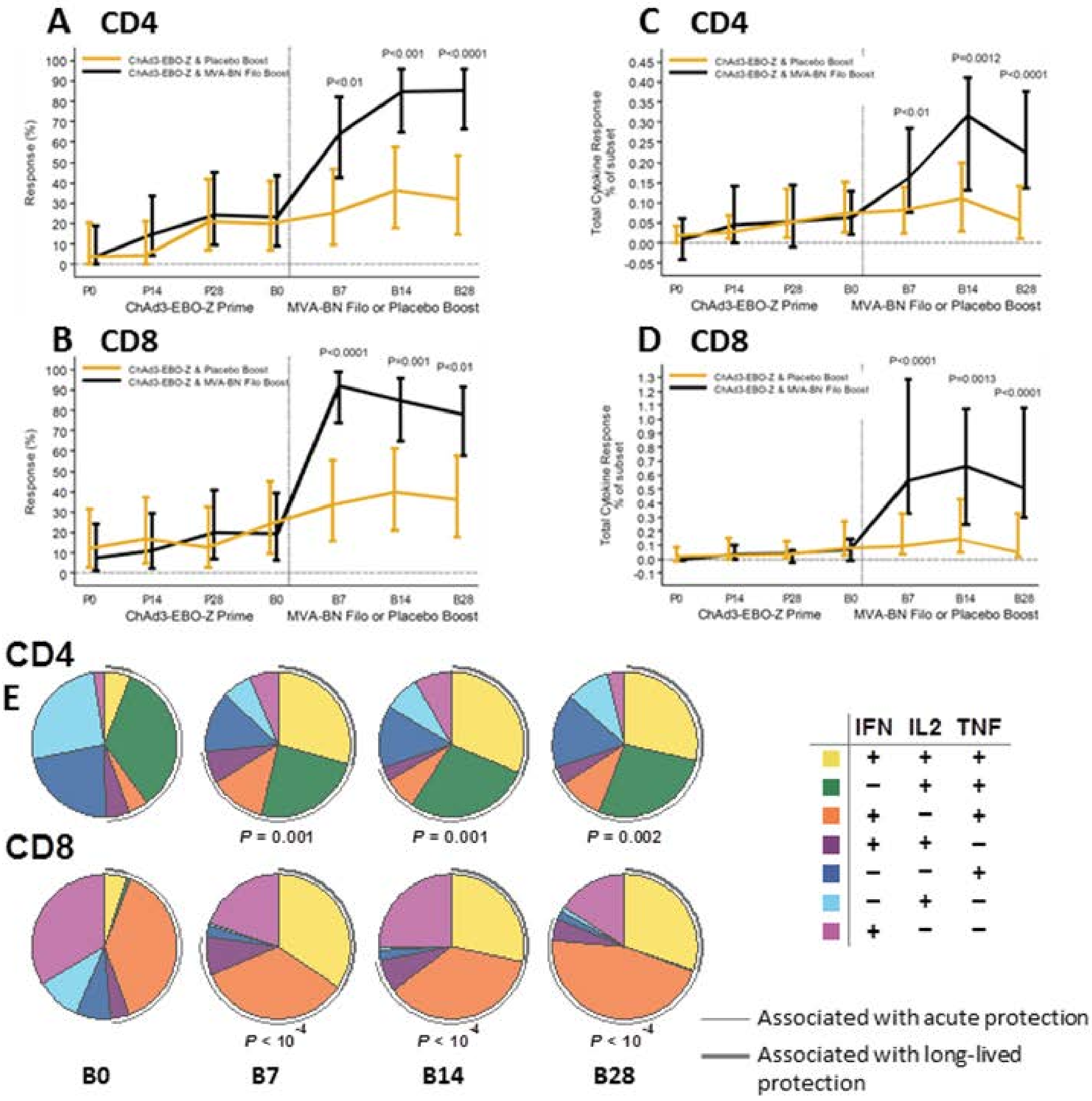

Supplemental Figure 5: The proportion of responders (%), magnitude of the memory CD4 and CD8 cytokine (IFN-γ, TNF-α and IL-2) responses stimulated by exposure of T-cells to peptide pools Z1 and Z2 from *Zaire ebolavirus* glycoprotein and pie charts summarizing multifunctionality of the responding memory T-cells.

Panels A & B. Percent of 52 participants vaccinated with ChAd3-EBO-Z vaccine who were subsequently boosted with MVA-BN®-Filo (N=27) or saline placebo (N=25), whose CD4 T-cells (Panel A) or CD8 T-cells (Panel B) exhibited significant responses upon stimulation with peptides from Zaire glycoprotein (Z1 or Z2 peptides). Days on the x-axis are displayed in relation to Priming immunization and Booster (P and B, respectively). P values compare responses observed in the sub-cohort boosted with MVA-BN®-Filo versus the sub-cohort that received placebo.

Panels C & D. Magnitude of the responses of 52 participants vaccinated with ChAd3-EBO-Z vaccine who were subsequently boosted with MVA-BN®-Filo (N=27) or saline placebo (N=25), whose CD4 (Panel C) or CD8 (Panel D) memory T-cells exhibited significant responses upon stimulation with peptides from Zaire glycoprotein (Z1 or Z2). Days on the x-axis are displayed in relation to priming immunization and Booster (P and B, respectively). P

values compare responses observed in the sub-cohort boosted with MVA-BN®-Filo versus the sub-cohort that received placebo.

Panel E. Pie charts displaying the multifunctionality of the responses upon stimulation with peptides (Z1 or Z2) from Zaire glycoprotein. Memory T-cells elaborating only one cytokine or combinations of two or three cytokines are identified by the colors in the insert.
Note -- Following ChAd3-EBO-Z priming vaccination, regardless of dosage, no association was found between the anti-glycoprotein ELISA response at Day 28 and either CD4 or CD8 response at Days 14 or 28. Similarly, among the 27 subjects who received the MVA-BN®-Filo **booster, no association was found between the anti-glycoprotein ELISA response 7 days post-boost and either CD4 or CD8 at any post-boost time point.**

Epilogue

ChAd3-EBO-Z vaccine testing consortium

Responding to the precipitous rise in Zaire Ebola virus disease in west Africa, in mid-August 2014, WHO assembled a consortium to accelerate clinical testing of ChAd3-EBO-Z monovalent Ebola vaccine.[1] Single-dose ChAd3-EBO-Z conferred 100% protection to NHPs against challenge with virulent Zaire Ebola virus that was lethal for 100% of controls.[2] Consortium institutions included: the Vaccine Research Center of the National Institute of Allergy and Infectious Diseases, whose scientists, collaborating with Okairos, developed ChAd3-EBO-Z and tested clinical specimens with antiglycoprotein ELISA; GlaxoSmithKline Vaccines, to scale up manufacture; Oxford University, pioneers in clinical testing of other chimpanzee adenovirus 3-vectored vaccines,[10] to initiate clinical trials expeditiously in the UK; the Center for Vaccine Development (CVD)–Mali, to perform phase 1b clinical trials in Mali; the Center for Vaccine Development of the University of Maryland School of Medicine (which, with the Malian Ministry of Health, jointly maintains the CVD–Mali enterprise) to do trials in US participants; the Wellcome Trust, to accelerate peer review and funding for Oxford phase 1 and Mali phase 1b trials; and WHO, which assembled the consortium and provided coordination.

The design of a phase 1b vaccine trial in Mali was driven by the public health emergency of epidemic Ebola virus disease in west Africa and the need to try to rapidly test candidate vaccines in clinical trials. The consortium was assembled by WHO to do clinical trials in North America, Europe, and west Africa to assess as expeditiously as possible the safety, tolerability, and immunogenicity of a single oral dose of ChAd3-EBO-Z monovalent Ebola vaccine. This consortium did not use a single common clinical protocol with the different sites functioning as components of a multicentre trial, but rather did individual but similar trials. The individual trial designs were drastically affected by initial availability of only a small number of doses of vaccine to be apportioned among the trials. Nevertheless, the schedule for collection of blood specimens for each study done by consortium members included common timepoints, and NS'

laboratory at the Vaccine Research Center of the National Institutes of Health served as a common reference laboratory where antibodies against Zaire Ebola virus glycoprotein were measured with ELISA in specimens from the multiple clinical trials done by the consortium.

Reference List

1. Levine MM, Tapia M, Hill AV, Sow SO. How the current west african ebola virus disease epidemic is altering views on the need for vaccines and is galvanizing a global effort to field-test leading candidate vaccines. J.Infect.Dis. 2015; 211:504-7.

2. Stanley DA, Honko AN, Asiedu C, Trefry JC, Lau-Kilby AW, Johnson JC, Hensley L, Ammendola V, Abbate A, Grazioli F, Foulds KE, Cheng C, Wang L, Donaldson MM, Colloca S, Folgori A, Roederer M, Nabel GJ, Mascola J, Nicosia A, Cortese R, Koup RA, Sullivan NJ. Chimpanzee adenovirus vaccine generates acute and durable protective immunity against ebolavirus challenge. Nat.Med. 2014; 20:1126-9.

3. Colloca S, Barnes E, Folgori A, Ammendola V, Capone S, Cirillo A, Siani L, Naddeo M, Grazioli F, Esposito ML, Ambrosio M, Sparacino A, Bartiromo M, Meola A, Smith K, Kurioka A, O'Hara GA, Ewer KJ, Anagnostou N, Bliss C, Hill AV, Traboni C, Klenerman P, Cortese R, Nicosia A. Vaccine vectors derived from a large collection of simian adenoviruses induce potent cellular immunity across multiple species. Sci.Transl.Med. 2012; 4:115ra2.

4. Sullivan NJ, Martin JE, Graham BS, Nabel GJ. Correlates of protective immunity for Ebola vaccines: implications for regulatory approval by the animal rule. Nat.Rev.Microbiol. 2009; 7:393-400.

5. Pine SO, Kublin JG, Hammer SM, Borgerding J, Huang Y, Casimiro DR, McElrath MJ. Pre-existing adenovirus immunity modifies a complex mixed Th1 and Th2 cytokine response to an Ad5/HIV-1 vaccine candidate in humans. PLoS.ONE. 2011; 6:e18526.

6. Mast TC, Kierstead L, Gupta SB, Nikas AA, Kallas EG, Novitsky V, Mbewe B, Pitisuttithum P, Schechter M, Vardas E, Wolfe ND, Aste-Amezaga M, Casimiro DR, Coplan P, Straus WL, Shiver JW. International epidemiology of human pre-existing adenovirus (Ad) type-5, type-6, type-26 and type-36 neutralizing antibodies: correlates of high Ad5 titers and implications for potential HIV vaccine trials. Vaccine. 2010; 28:950-7.

7. Thorner AR, Vogels R, Kaspers J, Weverling GJ, Holterman L, Lemckert AA, Dilraj A, McNally LM, Jeena PM, Jepsen S, Abbink P, Nanda A, Swanson PE, Bates AT, O'Brien KL, Havenga MJ, Goudsmit J, Barouch DH. Age dependence of adenovirus-specific neutralizing antibody titers in individuals from sub-Saharan Africa. J.Clin.Microbiol. 2006; 44:3781-3.

8. Quinn KM, Da CA, Yamamoto A, Berry D, Lindsay RW, Darrah PA, Wang L, Cheng C, Kong WP, Gall JG, Nicosia A, Folgori A, Colloca S, Cortese R, Gostick E, Price DA, Gomez CE, Esteban M, Wyatt LS, Moss B, Morgan C, Roederer M, Bailer RT, Nabel GJ, Koup RA, Seder RA. Comparative analysis of the magnitude, quality, phenotype, and

protective capacity of simian immunodeficiency virus gag-specific CD8+ T cells following human-, simian-, and chimpanzee-derived recombinant adenoviral vector immunization. J.Immunol. 2013; 190:2720-35.
